# Exploring the Sustainable Utilization of Deep Eutectic Solvents for Chitin Isolation from Diverse Sources

**DOI:** 10.3390/polym16223187

**Published:** 2024-11-16

**Authors:** Rou Li, Peng-Hui Hsueh, Siti Ayu Ulfadillah, Shang-Ta Wang, Min-Lang Tsai

**Affiliations:** Department of Food Science, National Taiwan Ocean University, Keelung 202301, Taiwan; kdv102307@gmail.com (R.L.); smj2b11@gmail.com (P.-H.H.); sitiayuulfadillah@gmail.com (S.A.U.)

**Keywords:** chitin, deep eutectic solvents, green processing, sustainable production

## Abstract

Deep eutectic solvents (DES) represent an innovative and environmentally friendly approach for chitin isolation. Chitin is a natural nitrogenous polysaccharide, characterized by its abundance of amino and hydroxyl groups. The hydrogen bond network in DES can disrupt the crystalline structure of chitin, facilitating its isolation from bioresources by dissolving or degrading other components. DES are known for their low cost, natural chemical constituents, and recyclability. Natural deep eutectic solvents (NADES), a subclass of DES made from natural compounds, offer higher biocompatibility, biodegradability, and the lowest biotoxicity, making them highly promising for the production of eco-friendly chitin products. This review summarized studies on chitin isolation by DES, including reviews of biomass resources, isolation conditions (raw materials, DES compositions, solid–liquid ratios, temperature, and time), and the physicochemical properties of chitin products. Consequently, we have concluded that tailoring an appropriate DES-based process on the specific composition of the raw material can notably improve isolation efficiency. Acidic DES are particularly effective for extracting chitin from materials with high mineral content, such as crustacean bio-waste; for instance, the choline chloride-lactic acid DES achieved purity levels comparable to those of commercial chemical methods. By contrast, alkaline DES are better suited for chitin isolation from protein-rich sources, such as squid pens. DES facilitate calcium carbonate removal through H^+^ ion release and leverage unique hydrogen bonding interactions for efficient deproteination. Among these, potassium carbonate-glycerol DES have demonstrated optimal efficacy. Nonetheless, further comprehensive research is essential to evaluate the environmental impact, economic feasibility, and safety of DES application in chitin production.

## 1. Introduction

### 1.1. Chitin

Chitin is a polysaccharide that contains nitrogen and comprises units of *N*-acetyl-D-glucosamine linked together in a linear fashion. It is considered a valuable natural polysaccharide [1,2]. Chitin possesses numerous beneficial properties that make it suitable for a wide range of applications across various fields. It is biodegradable, biocompatible, renewable, non-toxic, and hydrating, and it exhibits diverse biofunctionalities, such as anti-thrombogenic, homeostatic, immunity enhancement, and wound healing properties. Chitin can form films or fibers, can chelate heavy metal ions, is hydrophilic, and demonstrates a remarkable affinity for proteins [1,3,4].

#### 1.1.1. Chitin’s Structures

Chitin is a high molecular weight polymer consisting of *N*-acetyl-D-glucosamine, the monomeric unit of the chitin, linked by β-1,4 glycosidic bonds. The molecular formula of this amino monosaccharide is C_8_H_15_NO_6_ [5], which has two active hydroxyl groups (-OH) and one acetamide group (-NHCOCH_3_). Its structure contains numerous hydroxyl groups, carbonyl groups, and amine groups. In simple terms, when a chemical has a hydrogen atom connected to a highly electronegative element like nitrogen or oxygen, hydrogen bounding between the compounds are generated [6]. Hydrogen bonds affect physical and chemical properties and maintain the geometric shape of macromolecules (such as polysaccharides, proteins, etc.) [7]. Chitin’s structure exhibits strong intermolecular and intramolecular hydrogen bonding networks, particularly through the *N*-acetyl-D-glucosamine units, which can form robust hydrogen bonds between acetyl groups on the same or adjacent chitin chains [8]. Chitosan, a functional derivative of chitin, is formed by deacetylating the -NHCOCH_3_ group at the C-2 position of *N*-acetyl-D-glucosamine into -NH_2_, with a degree of deacetylation (DD) exceeding 51%. When exposed to an acidic environment, chitosan with a high DD degree have increased solubility. This solubility arises from the protonation of the -NH_2_ groups on the C-2 position of the repeating units of D-glucosamine, which turns the polysaccharide into a polyelectrolyte in acidic environments [9].

Chitin molecules create catenaries. The catenaries of each end, whether aligned in the same direction or not, are arranged in layers corresponding to three unique allomorphic forms: α-chitin, β-chitin, and γ-chitin. α-Chitin is rhombic in structure, and the adjacent layers are anti-parallel and have different directions; while, in β-chitin, it is monoclinic, and the adjacent layers are parallel and have the same direction. The γ-chitin in each third layer is orientated contrary to the prior two layers [10,11]. 

α-Chitin is the most prevalent structure of chitin, commonly found in the cell walls of yeast and fungi, shrimp shells, insect cuticles, and the shells and tendons of crabs and lobsters [12]. The prevalence of α-chitin crystals contributes to the stiffer characteristics of chitin fibrils, leading to high crystallinity, often reaching up to 80% [13,14]. The strong molecular interactions in α-chitin, including hydrogen bonds, van der Waals forces, ionic forces, and hydrophobic bonds, contribute to its molecular chains being entangled in a network-like structure. This structural arrangement confers α-chitin with better mechanical strength than β-chitin [15].

β-Chitin is frequently discovered in the endoskeleton of mollusks, such as squid pens, and is also present in the bodies of tube worms and diatom spicules [16]. β-Chitin has poorer intermolecular hydrogen bonding than α-chitin because of the parallel structure of its major chains. The usual crystallinity of the material is approximately 70% [13]. β-Chitin demonstrates greater reactivity in a variety of modification reactions when compared to α-chitin. This renders it more prone to chemical alteration, enzymatic breakdown, and thermal impairment while also exhibiting a more significant attraction to solvents [17,18,19]. The γ-chitin structure has a distinctive pattern where every third layer is oriented in the opposite direction compared to the two previous levels [20]. γ-Chitin shares similarities with both α-chitin and β-chitin, but its structure is more closely aligned with α-chitin. Initially, it was discovered in specific fungi, yeasts, and cocoons [11].

#### 1.1.2. Resources of Chitin

Chitin is found throughout the animal, fungal, and Protista kingdoms, spanning at least 19 animal phyla. Arthropods such as crustaceans (e.g., crabs, lobsters, shrimps), insects (e.g., wasps, bees, ants, beetles), arachnids (including spiders, scorpions, ticks, mites), as well as centipedes, millipedes, and various other groups, feature chitin in their biological structures. In addition, chitin is found in the cell walls of fungi (like mushrooms and yeasts), algae (including diatoms, coralline algae, and green algae), as well as some worms and tubular animals [21]. Different species have different proportions of chitin. It is worth noting that when species are closely related in terms of taxonomy, their behavioral responses are quite similar, and their chitin composition is also fairly comparable [22].

##### Crustaceans

The main source of chitin is the exoskeleton of crustaceans, such as crabs, lobsters, shrimps, prawns, krill, and crayfish. These crustaceans have shells composed mainly of minerals, chitin, and protein, each accounting for about 30% to 60%, 20% to 30%, and 20% to 40%, respectively; pigments and lipids account for up to 14% [23]. The exoskeletons of crabs and shrimps typically consist of three main layers: the epicuticle, the exocuticle, and the endocuticle [24]. The outermost layer, the epicuticle, is very thin and covers the skeleton’s surface. It was primarily composed of minerals, proteins, lipids, and small amounts of chitin. Beneath the epicuticle is the exocuticle, which is thicker and contains chitin–protein fibers along with calcite. The endocuticle is the innermost and typically thickest layer, consisting of multiple layers of chitin–protein fibers [25].

From a molecular standpoint, the long-chain polysaccharide chitin is arranged into fibrils to form the crustacean cuticle. The protein wraps around the fibrils to make chitin–protein fibers, which make them about 20 times wider. The fibers cluster into bundles, which are subsequently aligned in a parallel manner and create horizontal planes. The planes are arranged in a helical manner to form a twisted plywood structure. Following the initial plane, a portion of the twisted plywood structure rotates 180°, forming a Bouligand structure. Thus, it generates exocuticle and endocuticle repeatedly. The particular configuration plays a vital role in providing the cuticle with uniform mechanical characteristics in all directions [26]. However, the exocuticle has a complex twisted plywood structure, whilst the endocuticle has a thicker and rougher arrangement. The structural characteristics are crucial for imparting strength and flexibility and safeguarding the exoskeleton of crustaceans. The primary component of crab shells is the rougher endocuticle. On the other hand, prawn shells consist mostly of a more delicate exocuticle, which is partially transparent and fragile [27].

##### Mollusks

Chitin is also present in mollusks, particularly in squid pens [28,29]. Pens, also known as gladius, are internalized shells structurally similar to the heavily mineralized external shells. Squid pens are typically translucent, resilient, pliable, non-mineralized skeletal structures of chitin and proteins. The composition of squid pens varies depending on the species of mollusks and their growing environment. The composition of squid pens typically consists of approximately 43% to 75% proteins, 25% to 49% chitin, 0.2% to 0.8% ash, and 0.1% to 0.2% lipids [30,31]. Squid pens are composed of chitin and proteins, forming a durable core structure known as a chitin–protein complex. This complex is made up of β-chitin that is covered with protein and arranged in parallel patterns. Additionally, they contain chitin and mineral complexes, typically calcium carbonate (CaCO_3_), to offer mechanical strength for the organism. Furthermore, squid pens serve as the primary and crucial reservoir of β-chitin [32]. Each squid has only one pen, which accounts for just 1% of the squid’s total weight [30]. Despite being difficult to access, squid pens have a chitin content that reaches up to 40%. Furthermore, the chitin found in squid pens differs from that of crustaceans, making it a potentially valuable source of chitin [22].

##### Insects

Insects constitute the most abundant species in the world [33]. They are helpful for human nutrition since they provide proteins and peptides. After that, they have several biopolymers like silk and chitin, which are used in various industries and biotechnology applications [34,35]. Recently, the detrimental impact of insect species on agricultural crops, resulting in significant economic losses worldwide, has become increasingly apparent. These perilous species have the potential to be valuable suppliers of chitin, but they are not being fully utilized worldwide [36]. 

The evolutionary success of insects, classified as arthropods, can be attributed to the development of the cuticle, a multifunctional and intricate exoskeleton. Chitin plays a crucial role in the formation of the exoskeleton of insects. The chitin crystal structure facilitates both dense packing and great tensile strength due to the strong hydrogen bonding between chitin molecules [37]. Furthermore, chitin in the exoskeleton is frequently accompanied with CaCO_3_, a compound predominantly present in composite materials like sclerotin. In contrast to pure chitin, which possesses characteristics such as translucency, flexibility, durability, and toughness, this composite material is notably more rigid and resistant than pure chitin. Additionally, it is tougher and less prone to brittleness in comparison to pure CaCO_3_ [33]. On the other hand, the oxidation products of catecholamines help form strong interactions between chitin and protein fibers in the insect cuticle, leading to its solidification and providing the insects with a durable exoskeleton [38]. The exoskeleton provides structural support for muscle attachment during movement, acts as a barrier against physical and chemical harm, and offers protection against the spread of infectious diseases [37]. Chitin also provides structural support for the peritrophic matrices that cover the lining of the gut epithelium, as well as the cuticles of the trachea and epidermis [39]. 

Kaya et al. [40] suggested that the functions and composition of chitin can be affected by a range of parameters, including the type of insect, its growth stage, life cycle, body parts, and sex. The contents of chitin will be augmented by increasing the growth stage. The chitin contents of *Hermetia illucens* larvae in Greece and Portugal are 8.40% and 9.74%, respectively [41]. The chitin content in prepupae of *Hermetia illucens* was measured to be 10.9%, while in adults, it was found to be 8.4% [42]. Moreover, the properties of chitin will differ depending on the growth stage. The degree of crystallinity of chitin, derived from *Hermetia illucens*, increases progressively from larvae to adults. Specifically, the values are 33.09% for larvae, 35.14% for prepupa, 68.44% for pupa, and 87.92% for adults [43]. 

##### Fungi

Chitin can be obtained from non-animal sources, such as fungi [44], and is not affected by seasonal or regional variations, unlike chitin derived from crustaceans [45]. The cell walls of fungi are dynamic structures that are crucial for the survival, development, and disease-causing abilities of the cells [46]. However, not all fungi have chitin and chitosan. Chitin and chitosan are known to be present in the cell walls of fungi such as *Basidiomycetes*, *Ascomycetes*, *Zygomycetes*, and *Deuteromycetes* [47]. 

Fungi possess chitin in the structure of a chitin–glucan complex (CGC), consisting of chitin, β-(1,3) and β-(1,6)-glucan, mannan, and proteins. This complex is located in the core region of the fungal cell walls and consists of flexible, branching β-glucan combined with rigid chitin, leading to a nanocomposite structure with strong and resilient fiber networks [45,48]. Chitin has a high degree of polymorphism in fungal cell walls, with α- and γ-allomorphs present. The presence of chitin–glucan covalent linkages in fungal cell walls does not significantly alter the structural properties of chitin, suggesting that these connections may not play a crucial role in constructing the cell wall [49]. 

Mushrooms are the fruiting bodies of fungi. Furthermore, according to the FAOSTAT data report by the FAO, the global production of mushrooms and truffles witnessed significant growth from 495,127 tons in 1961 to 48.3 million tons in 2022 [50]. *Agaricus bisporus* accounts for 11% of global mushroom production and 97.6% of total production in the United States [51]. Mushrooms and their derivatives, particularly *A. bisporus,* often deteriorate rapidly. Due to the action of tyrosinase and melanin synthesis, they undergo quick transformation into darker substances with an awful odor, leading to disposal challenges in the environment [52].

Regardless of the fungal species or method utilized, the extraction of chitin from fungal species always begins with a dilute alkali treatment to remove proteins, glycoproteins, and branching polysaccharides in order to obtain alkali-insoluble material (AIM). Afterward, acid extraction isolates chitosan, whereas chitin and β-glucan retain alkali/acid insoluble residues [53,54]. Moreover, removing calcium carbonate, such as demineralization in crustacean chitin, is not required to purify fungal chitin [45]. 

Vetter [55] extracted 6.68% and 7.25% chitin from the pileus and stipe of *Agaricus bisporus*, respectively. However, the specific extraction process used was not detailed. Hassainia et al. [56] verified that stipes have a chitin content of up to 7.4%, while pileus and gills have chitin contents of 6.4% and 5.9%, respectively. According to Fadhil and Mousa [57], the dry weight percentage of chitin extracted from *A. bisporus* was 16%; and Wu et al. [53] reported crude chitin synthesis from *A. bisporus* stalks at 27% dry weight (0.65% to 1.15% fresh). The degree of acetylation (DA) of fungal chitin ranged from 75.8% to 87.6%, equivalent to commercial crustacean chitin. Chitin extraction yields vary depending on the mushroom fruit portion. *A. bisporus* stipes produced the greatest chitin (7.4% dry weight), followed by pileus and gills (6.4% and 5.9%, respectively). The extracted chitin was found to be in α-form, with a 63% crystalline index (CrI) and 70% DA [56]. In certain applications, *A. bisporus* chitin nanofibers outperform crab-derived chitin nanopaper in terms of translucency, hardness, and flexibility. The chitin nanopaper generated from *A. bisporus* has properties similar to standard nanopaper [58].

#### 1.1.3. Preparation of Chitin

Chitin is often regarded as one of the most abundant biomolecules found on Earth. Nevertheless, pure chitin is a rarity in nature and is closely combined with other substances. To obtain chitin, it is necessary to demineralize, deproteinize, and decolorize the raw materials [59]. Demineralization processes are mostly utilized to eliminate calcium carbonate (CaCO_3_) by employing hydrochloric acid (HCl), nitric acid (HNO_3_), sulfuric acid (H_2_SO_4_), acetic acid (CH_3_COOH), or formic acid (HCOOH). These acids react with the carbonate ion (CO_3_^2−^) to generate carbon dioxide (CO_2_), thus eliminating CaCO_3_. Dilute hydrochloric acid is the most frequently utilized reagent among all the options.

Protein removal processes can be categorized into chemical methods and biological methods. Chemical methods are more expensive, ecologically detrimental, and bring changes to the physicochemical characteristics of the chitin product. Nevertheless, chemical methods remain the predominant approach in the industry due to their high efficiency and rapid responsiveness [60]. The deproteinization process involves treating the substance with hot alkaline solutions, such as NaOH, Na_2_CO_3_, NaHCO_3_, KOH, K_2_CO_3_, Ca(OH)_2_, Na_2_SO_3_, NaHSO_3_, CaHSO_3_, Na_3_PO_4_, and Na_2_S. The reaction conditions for each alkali reagent vary significantly, with NaOH being a frequently employed reagent. Furthermore, NaOH has the ability not only to eliminate proteins but also partially deacetylate and hydrolyze chitin [12].

The primary objective of decolorization is to eliminate the inherent pigment present in the exoskeletons of diverse bioresources. The treatment can be performed using several techniques, such as the application of solvent extraction and redox agents. Solvent extraction is a procedure that entails dissolving pigments using solvents, such as acetone, chloroform, ethyl acetate, and ethanol. The pigments can be extracted concurrently by which the removal procedure was conducted [61]. In addition, utilizing an oxidizing agent and a reducing agent is useful in eliminating the undesired color of chitin products. Examples of potent oxidants that can be employed are potassium permanganate (KMnO_4_), either with or without hydrogen peroxide (H_2_O_2_), sodium hypochlorite (NaClO), phosphorus pentoxide (P_2_O_5_), sulfur dioxide (SO_2_), and sodium carbonate (Na_2_CO_3_) [33]. Most of the pigments are commonly extracted together with minerals and proteins. Decolorization is typically unnecessary unless excessive pigment remains [12].

### 1.2. Deep Eutectic Solvents

Deep eutectic solvents (DES) are a family of green solvents that feature characteristics of both ionic liquids and organic solvents [62]. DES are unique solvent systems in which the liquid mixtures have a melting point lower than any individual component. For instance, when urea (melting point = 133 °C) and choline chloride (melting point = 302 °C) are mixed in a molar ratio of 2:1, a eutectic mixture with a melting temperature of 12 °C is produced. The substantial decrease in melting point is attributed to the interaction between urea molecules and chloride ions [63]. Urea works as a hydrogen bond donor (HBD) by giving the lone pair of electrons from the hydrogen ion on -NH_2_; whereas choline chloride uses its chloride ions (Cl^−^) to act as a hydrogen bond acceptor (HBA). The hydrogen bonding resulting from the combination of choline chloride and urea decreases the amount of available energy in the mixed solution, forming a novel solvent with a decreased melting point [64].

DES can consist of multiple components, rather than just two, which are heated at different molar ratios and undergo self-combination by hydrogen bonding [65,66]. For easy understanding, DES composition is commonly abbreviated as Cat^+^X^−^zY, consisting of two single compounds. Cat^+^X^−^ is a single component in the DES system, where Cat^+^ can be any cation of ammonium salt, sulfates, and phosphates; X^−^ is a Lewis base, usually the anion of halogen salts; Y is another single component, usually a Lewis or Brønsted base; and z is the number of Y molecules corresponding to Cat^+^X^−^ [67]. DES are currently classified into four main types: quaternary salts and metal salts, quaternary salts and metal hydrates, quaternary salts and any compound that is an HBD, and metal chlorides and any HBD compounds [68,69]. 

The hydrogen bonding system of DES has a direct impact on its performance. The melting point decreases as the hydrogen bond network interaction increases, while the viscosity of the combination increases [70]. DES offer more straightforward and cost-effective preparation methods, more biodegradability, renewability, recyclability, and low toxicity, making them potentially eco-friendly [71]. However, further research is required to verify their environmentally beneficial features [72]. The DES selection component prefers organic quaternary amine salts, such as choline chloride or acetylcholine chloride as the HBA, due to their biodegradability and low toxicity. On the other hand, carboxylic acids, amides, and polyols are frequently chosen as the HBD for the same prospect [73,74]. Sharma et al. [75] showed that DES could effectively prepare or dissolve various biopolymers like lignin, cellulose, and starch. Vicente et al. [76] also reported that DES could dissolve chitin, allowing for a deacetylation process to produce chitosan.

The concept of green chemistry was raised in the 1990s, and the 12 principles of green chemistry, published by Anastas and Warner [77], provide a framework for scientific research and the manufacturing of eco-friendly products. The features of DES, such as their low toxicity, biodegradability, and adjustable viscosity, are in accordance with the 12 principles of green chemistry [78]. By enhancing efficiency, accelerating chemical processes, conserving energy, and serving as a regenerative solvent, DES possess the capacity to achieve sustainable development.

Rodrigues et al. [79] used choline chloride (ChCl) and varied lactic acid (LA), malic acid (MA), and malonic acid (MO) to make DES. They assessed the influence of DES on wheat (*Triticum aestivum*) seeds and determined the impact caused by DES on crops, which are important agricultural plants globally. Results indicated that ChCl–LA DES demonstrated the lowest phytotoxicity, followed by ChCl–MA and ChCl–MO DES. The EC_50_ values for germination inhibition were 11.8, 7.6, and 5.0 mg/mL, respectively. The EC_50_ values for shoot height inhibition were 1.6, 1.3, and 0.9 mg/mL, respectively. The findings suggested that the organic acid present in ChCl–organic acid DES significantly influenced the phytotoxicity of DES, potentially resulting in regulatory effects on the activity of antioxidant enzymes in wheat. The toxicity of germination or shoot and root growth was considered low as the EC_50_ was greater than 5 mg/mL [80]. Nevertheless, all DES are regarded as safe solvents since their EC_50_ values are higher than 1 mg/mL [81].

However, some DES still exhibit toxicity, which reduce their biocompatibility and limit their application in food and biomedicine [82]. Choi et al. [83] proposed the concept of natural deep eutectic solvents (NADES), a subtype of DES composed of natural chemicals. NADES comprise the common primary metabolites for cells, including choline derivatives, amino acids, monosaccharides, and organic acids that interact to each other through hydrogen bonding [84]. They possess outstanding solubility, low toxicity, biocompatibility, environmental friendliness, and sustainability. As a result, NADES have gained significant popularity in diverse areas, including chemical dissolution, separation engineering, and biocatalysis [85,86,87,88,89].

### 1.3. Objectives

Using the eco-friendly properties of DES and NADES aligns with the principles of green chemistry. Numerous study reports have documented the effective isolation of chitin utilizing DES, and several commendable review publications have been written [71,78,90,91]. This review seeks to validate DES as a viable method for chitin preparation and investigate the potential DES mechanisms between chitin isolation and the fundamental components of various chitin sources. This review paper is expected to facilitate the expedited comprehension and optimization of the DES chitin manufacturing process, consequently enhancing production efficiency.

## 2. Methodology

This study gathered all the pertinent literature on “deep eutectic acid” and “chitin” from the Web of Science database to fulfill the research objectives of this article. All studies that provide preparation methods and characteristic analysis for DES extraction or chitin separation are included in the scope of this study. In order to focus on the characteristics of direct preparation of chitin by DES, this article excludes 28 research studies concerning chitin preparation via enzymes, the application of DES for deacetylation, and the synthesis of chitin-related materials (e.g., membranes, nanofibers). Finally, this study selected 31 research articles for analysis, comprising 3 articles on chitin isolation from lobster shells, 11 articles on shrimp shells, 7 articles on crab shells, 3 articles on crayfish shells, 2 articles on insects, 2 articles on mushrooms, and 3 articles on squid pens, to elucidate and examine the composition of DES, the chitin preparation process, and potential mechanisms.

## 3. Chitin Preparation from Various Resources by DES

**Figure 1:**
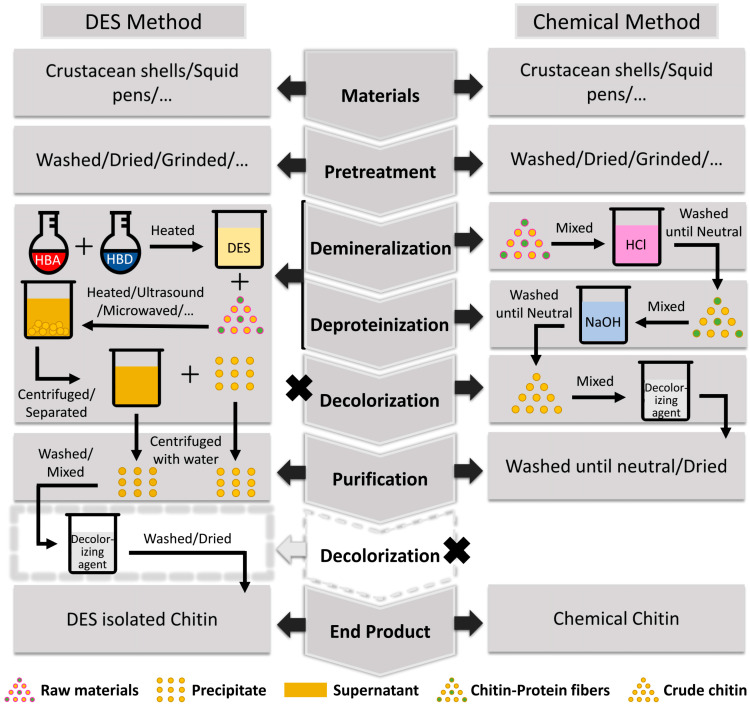
Process flow diagram of chitin preparation using DES and conventional chemical methods.

Multiple studies have utilized DES to prepare chitin from different biomass resources. This section will focus on several resources, such as lobster shells, shrimp shells, crab shells, crayfish shells, insects, mushrooms, and squid pens. This article provides a simple schematic diagram to compare chitin preparation procedures with conventional commercial chemical methods and the DES method (Figure 1). The DES method integrates demineralization and deproteinization phases into a single process, unlike chemical procedures that treat these steps separately. Due to DES’s ability to dissolve chitin [75], the product of the DES method can be categorized into supernatant and precipitate. Upon the addition of an antisolvent, predominantly water, to the supernatant, chitin will precipitate. Certain studies will examine the supernatant and precipitate independently; however, due to the often-poor yield of the supernatant (about 0.1% to 2.0%), most research focused on the precipitate as the primary subject or integrated both the supernatant and precipitate. Certain studies conduct supplementary decolorization following chitin preparation with DES, whereas others directly purify and dry the product for analysis. Since the pigment content in chitin resources is significantly lower than that of minerals and proteins, this review does not elaborate on decolorization but concentrates on a comprehensive introduction and discussion of demineralization and deproteinization. This review described the preparation methods, including DES component and molar ratio of the HBA and HBD, and the chitin preparation conditions, such as solid–liquid ratio, reaction temperature, and time. In this section, a detailed discussion will be conducted on the separation of chitin from bio-resources of various species using DES. In addition, the physicochemical characteristics of chitinous products obtained from various sources will be revealed. 

### 3.1. Lobsters

Zhu et al. [92] isolated chitin from lobster shells by using DES composed of ChCl and four specific chemicals (thiourea, urea (UR), glycerol (GY), and MO) with different molar ratios. The X-ray diffraction analysis (XRD) indicated that the α-chitin produced by ChCl–MO DES, with a molar ratio of 1:2, exhibited the highest purity. They treated lobster shells and DES using a 1:14 solid–liquid ratio (S/L) for 2 h at 50 °C. The chitin obtained via DES treatment was separated into supernatant and precipitant, which achieved yields of 4.44% and 16.19%, respectively, closely resembling the yield of 16.53% using the chemical method. The supernatant and precipitated chitin had crystallinity index (CrI) values of 67.2% and 80.6%, respectively, which are lower than the CrI of chitin prepared by the chemical method (82.5%). These findings indicated that the dissolution of chitin in ChCl–MO DES may result in the formation of amorphous chitin by breaking the hydrogen bonds that exist in chitin molecules. The examination conducted using a scanning electron microscope (SEM) demonstrated that the particle size of chitin in the supernatant was significantly smaller than that of precipitant or chitin obtained through the chemical method. The reduction in particle size may also suggest the fine dissolution of chitin in DES.

Hong et al. [93] investigated the process of isolating chitin from lobster shells using DES prepared by ChCl and four organic acids (MO, MA, LA, and levulinic acid) with different molar ratios. They treated lobster shells and DES using a 1:10 S/L ratio for 2 h at 50 °C, 70 °C, and 100 °C. The yields of the four DES separations ranged from 19.25% to 23.31%, superior to the chemical method, which yielded 17.21%. This suggests that the acidity of the HBD impacts the purity of chitin products. The chitin isolated by four different DES exhibited purity ranging from 90% to 93%, with the ChCl–MO DES showing the highest performance. The ChCl–MO DES isolation had a purity (93%) comparable to that of the chemical method and a lower quantity of protein and mineral residues. In addition, HBDs have a higher acidity, which may lead to the partial acid hydrolysis of chitin, decreasing its molecular weight (MW). For example, the MW of chitin produced from the chemical method was 546 kDa, and the MW of chitin obtained from ChCl–MA and ChCl–MO DES at 100 °C was 91 kDa and 199 kDa, respectively, confirming that MA is the most acidic of the four HBDs utilized. Lowering the reaction temperature reduces the extent of acid hydrolysis, which helps maintain the integrity of the chitin molecules. The CrI of α-chitin isolated using DES at 50 °C are all lower than 87.48% of the chitin obtained by the chemical method. Acidic DES induce the breakdown of hydrogen bonds in chitin molecules, leading to the formation of amorphous chitin and a decrease in crystallinity.

Zhu et al. [94] employed ChCl, LA, and four distinct polyols (ethylene glycerol, GY, xylitol, and sorbitol) to synthesize ternary DES with a molar ratio of 1:1:1. The results indicated that increased hydroxyl groups within the polyol structure correlated with an increase in hydrogen bond formation in DES, resulting in increased viscosity. This was due to a denser hydrogen bond network, which directly influenced the properties of DES, including viscosity and solubility. The separation efficacy of four varieties of ternary DES for chitin in lobster shells was evaluated with an S/L ratio of 1:20 at 50 °C for 2 h. The ChCl–LA–GY DES exhibited the most effective demineralization, with a residual ash content of 1.23%. The deproteinization effect showed no significant variation among the utilized DES, with protein residue ranging from 3.64% to 4.13%, which was also evident in purity and yield. The purity of commercial chitin and chitin produced by chemical method was 98.24% and 98.23%, respectively, and the yield of the chemical method was 16%. The chitin prepared by ChCl–LA–GY DES exhibited a purity of 94.76% and a yield of 26.22%. The physical and chemical characteristics were similar between chitin isolated by DES and obtained by chemical methods. ChCl–LA–GY DES separated α-chitin with a CrI of 77.73%, which is comparable to 80.58% for commercial chitin, and 78.78% for chitin produced by chemical method. The MW of the chitin prepared with ChCl–LA–GY DES was 351 kDa, lower than 706 kDa of chitin produced through chemical methods. It possessed a porous and dense fiber structure akin to commercial chitin.

In summary, DES can efficiently separate α-chitin from lobster shells, exhibiting comparable physical and chemical characteristics to traditional chemical methods. ChCl was presently recognized as the HBA. The HBD employed various organic acids, with malonic acid being frequently utilized. The optimal separation conditions indicated a molar ratio of 1:2 for DES composition, incorporating lobster shell powder at 50 °C for 2 h, with a S/L ratio of 1:10. The purity and MW of the chitin product will be influenced by the HBD acidity and reaction temperature, allowing for the separation of chitin with varying CrI. The investigation of different polyols in ternary DES for chitin production revealed that the separation efficacy was linked to the viscosity of DES and the number of hydrogen bonds present. An increased number of hydrogen bonds correlated with increased viscosity, influencing molecular motion. A decreased viscosity enhanced the efficiency of protein removal, and the purity of chitin separated via DES.

### 3.2. Shrimp Shells

Saravana et al. [95] employed ChCl and 14 distinct compounds to perform DES with a molar ratio of 1:2 and isolated chitin from the shells of kuruma shrimp (*Marsupenaeus japonicus*) with a 1:25 S/L ratio at 80 °C for 2 h. The yields of each DES exhibited significant variation, ranging from 21.52% to 75.92%. The chitin isolated with ChCl–LA and ChCl–MO DES exhibited the greatest purity and yields of 29.20% and 25.00% among all groups, respectively. Moreover, increased reaction time resulted in a corresponding decrease in protein and mineral residue amounts. The study prolonged the reaction time to 4, 8, 12, 24, and 48 h using ChCl–LA and ChCl–MO DES. After 48 h, the chitin yields by these two DES were 19.01% and 18.02%, respectively, higher than that by chemical method, which was 16.08%. Ultimately, the researchers used ChCl–MO DES to prepare α-chitin at 80 °C for 2 h. The MW was 79 kDa, and the CrI was 87.59%, slightly higher than the CrI of chitin obtained from the chemical method (86.99%).

Feng et al. [96] employed ChCl and five different organic acids (L–LA, L–citric acid (CA), L–MA, D–MA, and DL–MA) to treat *Solenocera crassicornis* shells. DES, which induces demineralization, deproteinization, and acylation, can produce *O*-acylated chitin straight from shrimp shells. The findings indicated that chitin was present in the supernatant of all DES treatments. However, yields were less than 1%. The ChCl–L–MA DES with a molar ratio of 1:2 had the highest chitin yield in precipitates, reaching up to 17.3%. It had an acyl substitution degree of 0.45 and a purity of 91.9%. MA exhibited the highest level of acidity compared to the other two organic acids. Although ChCl–L–MA DES have the highest deproteinization effect among the stereoisomers, it did not possess exceptional demineralization and acylation capabilities. The ChCl–DL–MA DES exhibited superior demineralization capability, yielding chitin with a purity of 92.6% and a yield of 27.2%. Consequently, ChCl–DL–MA was identified as the optimal DES in this case. The study examined the effects of temperature (90 °C to 150 °C), heating time (0.5 to 5 h), S/L ratio (ranging from 1:10 to 1:50), and water content (up to 20%) on the chitin straight acylation process. The optimum temperature for the reaction was 130 °C, and the purity improved since time or temperature increased. This may be attributed to the enhanced solubility of chitin in DES, facilitating fast acylation by interaction with hydrogen ions (H^+^) released by DES. The most effective S/L ratio was 1:20. The hydrogen bond network in chitin can be broken down quickly at a ratio of 1:50, which made the acylation process harder. Even though the chitin was very pure (99%), it had a low yield (4.6%) and a low degree of substitution (0.20%). Remarkably, ChCl–DL–MA DES with a molar ratio of 1:2 exhibited the highest yield of 15.9% and the lowest degree of substitution (0.49) at a water content of 10%.

Bradic et al. [97] synthesized DES using ChCl and four compounds (UR, LA, CA, and MO). Chitin was isolated from the shells of northern shrimp (*Pandalus borealis*) at temperatures ranging from 60 °C to 90 °C, using varying S/L ratios of 1:25 and 1:50 for 6 h. The findings demonstrated that ChCl–LA DES with a molar ratio of 1:1 had the highest yield (20%) and recovery yield (85%), due to the decreased viscosity of ChCl–LA DES compared to the other four DES that were utilized, and the viscosity decreased at high temperatures. Upon doubling the S/L ratio, the yields of each group exhibited a rise, while the purity remained consistently above 92%. When acidic ChCl–LA DES were exposed to a temperature of 70 °C, it produced α-chitin with a high MW of 125 kDa and a CrI of 91%. When alkaline ChCl–UR DES with a molar ratio of 1:2 was exposed to a temperature of 90 °C, it produced α-chitin with a low CrI (43%) and MW of 75 kDa. These findings indicated that ChCl–UR DES exhibited a significant ability to dissolve chitin at high pH and temperature. This resulted in the disruption of hydrogen bonds and the formation of partly amorphous chitin [75]. Chitin undergoes structural modifications, leading to chitin with decreased crystallinity and MW.

Feng et al. [98] produced chitin from *Solenocera crassicornis* shells with 1:20 S/L ratio at 70 °C for 3 h through ChCl with p-toluenesulfonic acid monohydrate (TsOH) to form ChCl–TsOH DES with a molar ratio of 1:2. At temperatures ranging from 70 °C to 110 °C, the results indicated that ChCl–TsOH DES effectively removed proteins and minerals and facilitated the dissolving of chitin and proteins at high temperatures. Nevertheless, 70 °C was selected as the best reaction setting due to the liquid state of ChCl–TsOH DES to remain at this temperature. Isolation with ChCl–TsOH DES with 15% water had a chitin purity of 97.9% and a recovery yield of 59.4%. The precipitated chitin had a CrI of 90.6%, similar to commercial chitin (90.4%). Once the water content approached 20%, the efficiency of protein removal decreased, but it did not affect demineralization. This phenomenon may occur due to association and hydration zones in the ChCl–TsOH DES/water system when the water content is below 15% and hydrated DES can release H^+^. Nevertheless, when the water content reaches 20%, the composition of the system undergoes a gradual transition from a fully bonded combination of ChCl and TsOH to partial hydration and eventually to complete hydration. This weakens the interaction between DES, protein, and CaCO_3_, preventing chitin dissolution and amorphous chitin regeneration. The researchers found that at reaction temperatures between 130 °C and 150 °C, the obtained supernatant could regenerate water-soluble carbon dots. Shrimp shells dissolved and decomposed in ChCl/TsOH DES, forming water-soluble compounds (carbon dots) and water-insoluble compounds (chitin).

Huet et al. [99] used ChCl–LA DES to separate chitin from *Crangon crangon* shells. The process was carried out at 110 °C, with an S/L ratio of 1:50 for 2 h. The results indicated that the ChCl–LA DES, with a molar ratio of 1:2, had lower effectiveness in removing minerals and proteins, resulting in a purity of 54.5% and a chitin recovery yield of 56%. The chitin purity could be increased by 6% with a treatment that combined DES with low-strength acid and alkali. This may be due to the removal of amorphous chitin in the materials through DES pre-treatment, that also resulted in an increase in the CrI of chitin products from 82% to 92%.

Sun et al. [100] prepared DES by combining ChCl with oxalic acid (OA), acetic acid (AA), LA, MA, and CA in a 1:1.5 molar ratio, followed by mixing shrimp shells at an S/L ratio of 1:20 and heating to 100 °C for 3 h. The use of ChCl–LA DES won the most optimal preparation outcomes. Since LA has the highest pKa value, increasing the proportion of LA enhances the yield efficiency. However, the protein removal efficacy diminished as the molar ratio approached 1:3, so the ultimate molar ratio of DES was adjusted to 1:2.5. Raising the reaction temperature to between 100 °C and 150 °C enhanced the mobility and collisions of molecules between DES and the target molecules, which further aided in reducing proteins and CaCO_3_ residues, leading to a higher purity of chitin. The DES treatment resulted in chitin with a purity comparable to conventionally produced chitin, reaching up to 99.33%. The chitin maintained its α-form structure and exhibited an acylation degree of 0.43. The introduction of acetyl groups likely disrupted hydrogen bonding inside and between chitin molecules during the acylation process. This disruption produced a CrI of 80.32% for DES-treated chitin, which was lower than that of commercial chitin (85.49%).

Zhang et al. [101] synthesized DES by mixing ChCl and GY in a molar ratio of 1:2. Shrimp shells were initially blended with DES at an S/L ratio of 1:29 and heated at 100 °C for 3 h. Various weight percentages of AA, ranging from 2.5% to 10%, were tested at 100 °C for 1 h, followed by treatment at temperatures ranging from 80 °C to 140 °C. It was determined that adding 7.5% AA and reacting at 120 °C produced chitin with 96.1% purity, 1.1% crude protein, and 0.4% ash content. However, the yield decreased from 27.4% to 21.4% after decolorization with 0.5% NaClO. Additionally, higher temperatures and AA concentrations resulted in a decrease in chitin yields due to chitin degradation. As an illustration, the MW of chitin treated with 2.5% AA was 270 kDa, but the MW of chitin treated with 10% AA was 190 kDa. Nevertheless, the degree of acetylation (DA) of each chitin product, commonly considered vulnerable to degradation by acid or alkali, remained consistently over 80%. Under optimal conditions with a 7.5% AA, the MW of chitin was 228 kDa, greater than that obtained by using the chemical method (218 kDa). The chitin generated by ChCl–GY DES with 7.5% AA demonstrated a denser crystal structure, as indicated by a CrI of 84.3%, significantly exceeding the CrI of 75.1% obtained by the chemical method.

Lei et al. [102] separated chitin from shrimp shells with a 1:20 S/L ratio at 80 °C for 2 h using DES composed of ChCl and various organic acids (MA, LA, CA, tartaric acid (TA), and OA) in 1:1, 1:2, and 1:3 molar ratios. The results suggested that, except for ChCl–LA DES, the other DES remained turbid during the separation procedure but could still effectively deproteinize and demineralize the chitinous materials. The ChCl–TA DES with a molar ratio of 1:3 exhibited outstanding quality, characterized by a purity of 87.73% and proteins and ash residues of 11.26% and 1.01%, respectively. The capacity of MA, CA, or TA to denature proteins and bind with ChCl or the HBD may explain the creation of a novel DES system. As the proportion of HBD increased, the deproteinizing capacity of DES also increased. However, in the ChCl–LA DES system, increasing the amount of LA reduced the deproteinizing capacity. It is thought that LA is ineffective at removing proteins that are strongly bound to chitin and instead relies on the hydrogen bond network of DES. Furthermore, the ChCl–LA DES (at a molar ratio of 1:1) did not cause chitin acylation, contrary to previous studies [96,100]. This could be attributed to the reaction occurring at a relatively low temperature of 80 °C and the presence of multiple hydrogen bonds in the DES system. These hydrogen bonds prevent the release of free H^+^, which inhibits the chitin acylation process. ChCl–OA DES had outstanding deproteinization efficacy, rapidly compromising the integrity of protein–chitin fibers. However, it also rapidly reacts with calcium ions to generate calcium oxalate, forming precipitates. All four groups, except ChCl–OA DES, produced α-chitin with a CrI greater than 95%.

Huang et al. [103] employed a 1:1 molar ratio of ChCl–MA DES to separate chitin from shrimp shells by microwave irradiation. They also investigated the impact of different S/L ratios (1:5, 1:10, and 1:20) as well as varying microwave times (1, 3, 7, and 9 min) on the separation of chitin. The findings indicated that when the S/L ratio was 1:20 and the microwave treatment time was 9 min, the rates of demineralization and deproteinization could reach 99% and 93.8%, respectively. The removal rate improved as the S/L ratio and the microwave time increased. The α-chitin obtained through DES exhibited a higher CrI (70.91%) compared to that obtained through the chemical method (65.41%).

Zhao et al. [104] isolated chitin from shrimp shells using a two-step method. Initially, the shrimp shells were pretreated with 10% CA, resulting in a demineralization rate of 98.15%. Subsequently, four types of DES, including betaine–UR (Bet–UR, ChCl–UR, ChCl–ethylene glycol, and ChCl–GY, were employed under different S/L ratios (1:5, 1:10, 1:15, and 1:20) and heated by microwave irradiation for varying times (1, 3, 5, 7, and 9 min). The optimal parameters were an S/L ratio of 1:20 and 7 min of microwave time. The rate of deproteinization was raised with the increase of both the S/L ratio and microwave time, with all types of DES achieving deproteinization efficiencies between 88.6% and 93%. Chitin yields varied from 22.5% to 25.1%, surpassing the yield achieved by the chemical method, which was 17.7%. In addition, the DA of chitin obtained by DES varied from 91.3% to 95.1%, significantly higher than the 86.12% DA achieved by the chemical method. This study illustrated that DES inflicted less damage on the acetyl group of chitin than strong alkali, which was prone to acetyl removal under high-temperature conditions. The MW of chitin obtained by DES varied from 290 kDa to 370 kDa, higher than the 250 kDa obtained via the chemical method, indicating that microwave-assisted DES isolation minimized excessive chitin degradation. Each DES type exhibited the following CrI performance: Bet–UR had 70.8%, ChCl–UR had 81.0%, ChCl–ethylene glycol had 80.8%, and ChCl–GY had 69.5%, all of which exceeded the 65.4% CrI of the chemical method. This suggests that the chemical method, using HCl and NaOH, may cause chitin swelling and an increase in the crystal plane distance, which lowers the overall crystallinity.

He et al. [105] developed a novel ternary DES by combining *N*-methylacetamide (MLA), *N*-methylurea (MU), and AA in a molar ratio of 1:1:3, abbreviated as MLA–MU–AA DES. This was subsequently used, along with microwave treatment, to isolate chitin from whiteleg shrimp (*Litopenaeus vannamei*) shells. The results showed that extending the microwave time and raising the S/L ratio improved the rate of both deproteinization and demineralization. The optimal conditions were an S/L ratio of 1:30 and a microwave time of 11 min, resulting in a 96.74% deproteinization rate and a 94.29% demineralization rate. MLA–MU–AA DES were also capable of isolating chitin at room temperature. After 48 h, the demineralization rate was determined to be 99.07%, while the deproteinization rate was 92.67%. The CrI of the chitin obtained at room temperature was 82.83%, similar to the 85.83% CrI of chitin obtained using the traditional chemical method. The CrI of chitin produced after DES and microwave treatment was 73.86%. This could be due to the shorter interaction time between chitin and AA during microwave treatment compared to the room temperature process, which allows only limited hydrolysis in the amorphous regions of chitin. As a result, the acetyl groups and the structural integrity of the chitin molecules are better preserved. The MW of chitin obtained by DES and microwave treatment was 1240 kDa, which exceeded the 909 kDa obtained through the chemical method and the 837 kDa obtained through DES treatment at room temperature.

Multiple studies have demonstrated the efficacy of DES in isolating chitin from shrimp shells. Organic acids have been found to have a critical role in the process of chitin separation. ChCl is utilized as the HBA and interacts with different HBDs to produce NADES, which is consistent with the principles of green chemistry [95,96,97,100,102]. Even when using neutral or alkaline NADES as solvents for separation, organic acids are added before or after the process to enhance the efficiency of isolation [101,104]. The chitin isolated from shrimp shells via DES treatment had unique properties compared to chitin produced through the chemical method. These attributes encompass increased crystallinity [95,98,99,102,103], molecular weight [97,101,104,105], and acyl substitution degree [96,100].

### 3.3. Crab Shells

Rodrigues et al. [79] used ChCl and varied LA, MA, and MO, respectively, to make DES. Brown crab (*Cancer pagurus*) shells and DES were blended at a 1:25 S/L ratio at different temperatures (50 °C, 80 °C, and 130 °C) for 2 and 4 h. After a 2-h reaction time, water was continuously added, and the mixture was stirred until it cooled to room temperature. This process dramatically enhanced the rate of demineralization in all groups by approximately 1.5-fold. This improvement can be attributed to two factors. First, water disrupts the DES system, causing previously dissolved chitin to precipitate out. Second, water promotes the formation of charged species, such as H_3_O^+^, which results in an acidic environment that promotes interactions between acids and minerals. However, adding water also slowed the rate of deproteinization in all groups. To address this problem, researchers dried samples and treated them with hydrogen peroxide (H_2_O_2_) to complete the deproteinization process. The ChCl–LA DES exhibited the highest deproteinization rate, achieving 100% deproteinization. Additionally, it produced α-chitin with 98.2% purity, 98.5% DA, and 82.9% CrI. These results were similar to those obtained by the chemical method, which prepared chitin with a DA of 98.0% and a CrI of 85.7%. The ChCl–LA DES had been recognized as the optimal option for chitin preparation.

Huang et al. [106] utilized ChCl–MA DES (with a molar ratio of 1:1) to separate chitin from crab shells using microwave irradiation. The demineralization and deproteinization rates will increase as the S/L ratio and microwave time increase. When microwaves were used with an S/L ratio of 1:30 and a duration of 11 min, α-chitin was isolated with a demineralization rate of 99.8% and a deproteinization rate of 92.3%. The structure and SEM data were similar to those of chemically isolated chitin.

Wang et al. [107] utilized gluconic acid (GA) and 19 distinct amino acids to develop several innovative DES for the separation of chitin from the shells of snow crab (*Chionoecetes opilio*). The purity of chitin products increased as the reaction temperature, time, and S/L ratio increased within the range of 60 °C to 110 °C, 6 to 24 h, and 1:10 to 1:50, respectively. At the same time, the yields decreased due to lower impurity residuals. Optimal efficiency was achieved at 100 °C for 6 h with an S/L ratio of 1:20. GA–cystine (GA–Cys) DES also had the highest chitin solubility, reaching 295 mg/g at 90 °C. It also provided the best separation conditions, producing chitin with a purity of 94.5% and a recovery yield of 79.1%. This recovery yield was 1.35 times higher than the chemical method, which achieved only 58.7%, although the chemical method produced slightly higher purity chitin at 95.6%. The GA–Cys DES strategy yielded α-chitin with a CrI of 74.9%, much higher than the chemical method (30.6%). Its MW was 375 kDa, which was 3.02 times higher than the MW of chitin produced via chemical methods (124 kDa). This implied that GA–Cys DES may reduce chitin degradation during isolation compared to traditional chemical methods.

Wang et al. [82] utilized two HBAs (betaine (Bet) and ChCl), along with six HBDs (*N*-acetyl-D-glucosamine (AG), D-GA, 5-hydroxymethylfurfural, levulinic acid, AA, and formic acid (FA)) to perform binary DES. Chitin was separated using DES with an S/L ratio of 1:20 at 130 °C for 3 h from snow crab (*Chionoecetes opilio*) shells. The results showed that ChCl had a stronger isolation effect than Bet. This could be due to Bet–HBDs DES’s high viscosity, which hinders the system’s diffusion of heat and chemicals. Increasing the acidity of the HBD resulted in higher chitin purity but lower recovery yield, which may also cause chitin degradation. When the binary ChCl–FA DES were used under optimal conditions, it produced chitin with a purity of 93.4% while chitin acylation proceeded. Thus, the authors created a ternary DES (ChCl–AG–FA) by combining AG, ChCl, and FA with a molar ratio of 1:0.6:1.4. The results indicated that ternary DES enhanced both the purity and recovery yield compared to binary ChCl–AG and ChCl–FA DES. This could be attributed to AG, a constituent of the chitin polymers, which can reduce acid degradation and damage toward chitin molecules, hence improving the recovery yield without affecting the purity. Furthermore, increasing the reaction temperature from 90 °C to 130 °C resulted in higher purity but lower recovery yield. However, the recovery yield plateaued at 130 °C, and increasing the temperature to 150 °C only raised the purity by an additional 2.4%. Similarly, extending the processing time from 3 to 5 h increased the purity by 1.6% while decreasing the recovery yield by 3.7%. The S/L ratio went from 1:5 to 1:50, purity rose from 74.6% to 94.3%, and recovery yield fell from 91.6% to 78.3%. Increasing the water content (0% to 50%) resulted in lower purity but higher recovery yields. For example, with 10% water, purity decreased by 7.2%. Finally, the authors used ChCl–AG–FA DES at an S/L ratio of 1:20 and 130 °C for 3 h. The process produced α-chitin with 90.2% purity and 85.6% recovery yield. The CrI and MW were measured at 52.6% and 392 kDa, respectively. This showed that ChCl–AG–FA DES had a gentler separation approach than the chemical method, which had a CrI of 30.6% and MW of 124 kDa.

McReynolds et al. [108] investigated the use of ChCl–MO and ChCl–LA DES to produce chitin from Henslow’s swimming crab (*Polybius henslowii*) shells. The chitin was manufactured at an S/L ratio of 1:25, and the process was carried out at temperatures ranging from 50 °C to 120 °C for either 1 or 2 h, respectively. Research revealed that increasing both the temperature and duration enhances production efficiency. The chitin produced using the chemical method acted as a control, resulting in a yield of 12.9% and a nitrogen concentration (%N) of 6.4%, as measured by elemental analysis. Thomas et al. [109] reported that the chitin with a DA range from 50% to 100% had a %N between 6.0% and 6.9%. After being heated to 120 °C for 2 h, the chitin gave results similar to chemically obtained chitin. The chitin was produced with ChCl–MO DES and had 6.9%N and a yield of 12.0%. By contrast, the ChCl–LA DES produced chitin with a %N of 6.8% and a yield of 12.8%. This could be attributable to the high viscosity of DES at low temperatures, which prevents acidic DES molecules from effectively entering the chitin–protein fiber, leaving the protein ineffective for removal. When comparing the chemical procedures and ChCl–MO and ChCl–LA DES isolation, it is seen that ChCl–LA DES-prepared chitin exhibited a greater DA at 94.1% and CrI at 81.9%. In addition, the authors claimed that they identified an acetyl group in chitin via DES separation, which was unique from chemical chitin. The reason could be attributed to the robust interactions between DES and macromolecules, such as hydrogen bonds, which pose challenges for total elimination. This offers a different perspective from previous investigations.

Wang et al. [110] chose two DES, ChCl–LA and ChCl–MA, and substituted a portion of the HBD with GY to create a novel ternary DES to separate chitin from snow crab (*Chionoecetes opilio*) shells with a 1:20 S/L ratio at 80 °C for 2 h. The results indicated that when GY was used to substitute certain acids, the isolation efficiency, chitin yield, and purity were unchanged. The CrI of α-chitin produced by each group exceeded that of chemically derived chitin (81.62%). Chitin from the MA series DES exhibited greater crystallinity and a more porous structure compared to that from the LA series DES. This is likely since MA is a tribasic acid, which creates a more stringent acidic environment and greater penetration ability than LA. Consequently, the chitin isolated from MA series DES had a higher porosity on SEM. The acid hydrolysis capacity of ternary DES diminished when GY was substituted. As a result, the MW of chitin in the LA series DES increased as the GY ratio increased. However, the ChCl–MA–GY DES, with a molar ratio of 2:1:1, had a greater proportion of MA, leading to chitin isolation with a reduced MW. Chitin produced under these conditions may contain residual impurities, resulting in reduced purity and affecting the characteristics of chitin.

Ma et al. [111] employed triethylbenzylammonium chloride (TEBAC) as the HBA and LA as the HBD to perform DES at various molar ratios of HBAs to HBDs for the separation of chitin from snow crab shells. The FTIR spectra of DES produced at molar ratios of 1:1 and 1:27 were studied, revealing that DES at both ratios exhibited similarities to LA. The C-H locations validated the presence of TEBAC, and the shift of the -OH group position of LA suggested that DES could be effectively produced using varying molar ratios. The demineralization rate indicated that an increased ratio of LA (HBA:HBD = 1:9 to 1:36) correlated with a higher demineralization rate, peaking at a molar ratio of 1:36. The removal rate was 95.49%, perhaps due to LA’s capacity to eliminate substantial quantities of CaCO_3_. The deproteinization rate escalated with an increasing ratio of LA (HBA:HBD = 1:9 to 1:27) and subsequently diminished (HBA:HBD = 1:36), with the maximum deproteinization rate reaching 84.17% at a molar ratio of 1:27. This may occur because the HBD initially experiences demineralization with CaCO_3_, compromising the internal structure of the crab shells and resulting in the dispersion of chitin–protein fibers in DES to extract proteins. This yielded the highest chitin purity of 84.58% at a molar ratio of 1:27, which was designated as the ideal molar ratio for further investigation. The influence of reaction temperature (110 °C to 130 °C) on chitin separation was examined. The findings indicated that the rates of demineralization and deproteinization escalated with rising reaction temperatures. Nevertheless, given that the boiling point of LA is 122 °C, 120 °C was ultimately chosen as the optimal reaction temperature. The impact of reaction time (2 to 6 h) on chitin separation was investigated. The results indicated that the maximal demineralization rate was 96.41% at 6 h, whereas the deproteinization rate reached its maximum value of 88.94% after 4 h. Consequently, 6 h was determined to be the best reaction duration. The impact of the S/L ratio (1:10 to 1:40) on chitin separation was examined. The findings indicated that it had a substantial effect on the deproteinization rate. The optimal deproteinization rate was 95.51% at an S/L ratio of 1:40, which did not influence the demineralization rate. This may be due to the LA in DES, which is capable of eliminating the crystalline CaCO_3_ in the crab shells, disrupting the internal structure of the crab shells, and facilitating total protein extraction in the designated S/L ratio. The isolated chitin product had a structure analogous to commercial chitin and was identified as α-chitin. With the rise in temperature and reaction duration, acylated chitin was produced. The S/L ratio of 1:40 and 1:20 exhibited higher CrI than commercial chitin (77.84%), with a 1:40 S/L ratio demonstrating the greatest CrI. This may be attributed to the removal of the amorphous region of chitin during the separation process, allowing for a greater reaction surface with DES, hence resulting in an elevated CrI. SEM observations indicated that chitin with an S/L ratio of 1:20 outperformed commercial chitin. Chitin exhibited bigger pores and clearer fibers, substantiating that DES could efficiently eliminate proteins and minerals from crab shells. 

Due to the similarity in the basic components of crab and shrimp shells, DES’s isolation conditions and features show similar optimal conditions and product characteristics. Wang et al. [107] developed several GA–amino acid DES to highlight their exceptional effectiveness in producing crab chitin. These DES also exhibited low phytotoxicity [79], making them crucial for environmental preservation and green environmental protection initiatives. In Ma et al. [111], TEBAC was identified as the HBA, and a comparison with various molar ratios of the HBD indicated that TEBAC–LA DES possess a structure analogous to LA. With the increase in the molar ratio of LA, the rates of demineralization and deproteinization also escalated, indicating that high-purity chitin can be more readily obtained through the use of acidic DES [106,108]. Furthermore, by partially substituting the acidic HBD, the depolymerized and acylated capacity of DES were diminished, allowing for the acquisition of chitin with a higher MW or a lower degree of acylation [82,110]. In addition, reaction temperature and duration were significant variables in separating chitin from crab shells. Nearly all investigations demonstrated that higher temperature and duration resulted in enhanced chitin purity [82,107,108,111]. This may result from the reduced viscosity of DES at higher temperatures, which facilitates an increased reaction surface area and rate, thereby enhancing separation efficiency [108].

### 3.4. Crayfish Shells

Bisht et al. [112] discovered that ChCl–LA or Bet–LA DES with a molar ratio of 1:2 may dissolve 10% purified commercial chitin at 115 °C for 20 h. These two DES can be used to isolate chitin straight from crayfish shells with an S/L ratio of 1:20 at 115 °C for 20 h. The yield of chitin reached 18%, and the recovery yield was 85%. Its physicochemical characteristics were similar to commercial chitin. For example, the DA of ChCl–LA DES and Bet–LA DES obtained chitin vs. commercial chitin were 93%, 96%, and 95%, respectively. The CrI of chitin produced via ChCl–LA and Bet–LA DES compared to commercial chitin was 83%, 85%, and 88%, respectively. Furthermore, isolated chitin showed the same CrI as pure chitin dissolved in DES, demonstrating that chitin was structurally intact. The MW of ChCl–LA and Bet–LA DES treated chitin was 194 kDa and 224 kDa, respectively.

Li et al. [113] used ChCl to make DES that included LA, GY, and UR with a molar ratio of 1:10. These solvents were used with microwave assistance to produce chitin from crayfish shells at 120 °C. The chitin isolated by ChCl–GY DES had purity, yields, and recovery yields of 41.99%, 88.17%, and 53.40%, respectively. The chitin isolated by ChCl–UR DES was 45.97% pure, yielded 88.87%, and recovered 49.16%. The protein residual percentages were 13.53% and 1.62%, whereas the ash residual percentages were 56.48% and 52.41%, respectively. The ChCl–LA DES produced chitin with a purity of 97.44%, yields of 19.11%, recovery yields of 73.22%, residual protein content of 2.56%, and an almost negligible ash residue. The optimal preparation solvent identified was ChCl–LA DES. The α-chitin produced demonstrated a crystallinity index (CrI) of 86.16%, which is slightly higher than the 82.63% observed in commercial chitin. Furthermore, elevating the molar ratio of LA in DES (1:1 to 1:10), the S/L ratio (ranging from 1:5 to 1:30), the microwave temperature (ranging from 80 °C to 140 °C), and the time (ranging from 10 min to 40 min) lead to increased chitin purity but decreased yields and recovery yields. At a ChCl:LA molar ratio of 1:10 and S/L ratio of 1:10, and microwave at 120 °C for 30 min, the generated chitin reached a state of high purity that no longer varied rapidly. Their economic cost-effectiveness determined the selection of the most efficient preparation methods. Surprisingly, the yields and recovery yields increased when the heating period exceeded 30 min. This may be due to the fact that, after 20 min, most of the minerals and proteins present in the shells had been removed. Chitin acylation took place after 20 min. The ongoing production of acylation chitin leads to greater quantities and enhanced processing efficiency.

Zhang et al. [114] conducted a study on ternary DES to separate chitin from crayfish shells. They first mixed selected serine (Ser) and proline (Pro) with urea in a molar ratio of 1:2, respectively, and then mixed with different weight percentages of 1,8-diazabicyclo [5.4.0] undec-7-ene (DBU). The mixture was agitated at 110 °C to produce a ternary DES. DBU possesses a notable capacity to capture protons, allowing it to capture carboxyl protons from amino acids. As a result, DBU becomes a positive charge while the amino acids become a negative charge. Urea possesses carbonyl and amino groups, which can easily react with amino acids and DBU to form the stable network structure of DES. The researchers employed deproteinization by utilizing Pro–UR/75% DBU DES, which exhibited the maximum chitin solubility at 6.0%. To obtain pure chitin, all crude chitin containing minerals from the DES process must be demineralized with a 20% LA solution for 1 h at 50 °C. The α-chitin obtained from crayfish shells with an S/L ratio of 1:20 at 110 °C for 20 h exhibited a purity of 91.39% and a recovery yield of 89.23%. It had DA of 65%, degree of substitution of 0.31, CrI of 78.85%, and MW of 236 kDa. The deproteinization rate was 97.31% and positively correlated with the percentage of DBU proportion, reaction time, and S/L ratio. This may be because alkaline DES have a more effective protein removal effect. An unfavorable consequence is the partial deacetylation of chitin at high temperatures, leading to a reduced DA of chitin isolated with DES compared to commercial chitin (92%). As the DBU% increased, the CrI fell, which can be attributed to chitin acetylation. The introduction of side chain groups into the structure disrupts the chitin molecules’ original tightly bonded hydrogen network. Furthermore, Pro–UR/75% DBU DES had a demineralization rate of only 3.98%. On the other hand, Pro–UR/75% DES with 20% LA separation yielded chitin with purity of 92.28% and recovery yield of 90.12%; calcium lactate was formed as a byproduct.

DES has been shown to separate chitin from crayfish shells, and prior research has shown that acidic DES were more conducive to the manufacture of chitin comparable to commercial chitin [102,112]. However, basic DES, such as the ternary Pro–UR/75% DBU DES [114], had strong deproteinization capabilities, enabling the production of high-quality chitin products through organic acid post-treatment.

### 3.5. Insects

Zhou et al. [115] employed two different types of HBAs (Bet and ChCl) and five different types of HBDs (LA, butyric acid (BA), OA, GY, and UR) to synthesize DES for the purpose of separating chitin from skimmed prepupae of black soldier fly (*Hermetia illucens*). Except for ChCl–UR, which had a molar ratio of 1:1, the other DES had a molar ratio of 1:2. This process was conducted at 50 °C or 80 °C for 2 h with an S/L ratio of 1:10. Upon researching 10 different types of DES, it was shown that ChCl–OA and Bet–OA DES could not be measured in pH due to their solid state at room temperature. Acidic DES were arranged in increasing pH order (0.16 to 5.79) as follows: ChCl–LA, ChCl–BA, ChCl–GY, Bet–LA, Bet–BA, and ChCl–UR. The neutral Bet–GY DES had a pH of 7.51, while alkaline Bet–UR DES had a pH of 9.58. The results showed that the pH of DES were not correlated with the chitin product’s purity, yields, or DA. However, there was a correlation between pH and the rate of demineralization, deproteinization, and CrI. Regardless of whether they were treated at 50 °C or 80 °C, ChCl–LA and Bet–UR DES demonstrated the highest rate of demineralization and produced exceptionally pure chitin. Interestingly, despite being solid at room temperature, acidic ChCl–OA and Bet–OA DES still demonstrated excellent demineralization rates, since the structure of OA includes two carboxyl groups with pK_a1_ = 1.36 and pK_a2_ = 4.11. When OA was substituted with BA, a similar structure in that an ethyl group replaced the carboxyl group of OA, resulting in a decrease in demineralization capability. Furthermore, temperature affects the acidity of DES, reducing the interactions between the HBA and the HBD and facilitating the interaction between electron withdrawing groups, such as carboxyl groups and biomass components, which enhances the efficiency of the preparation process and establishes a direct relationship between pH value and demineralization rate. At 80 °C, Bet–UR DES outperformed the chemical approach in terms of deproteinization rate. This implied that the rate of deproteinization was positively correlated with temperature. Across a temperature range of 50 °C to 80 °C, seven different DES showed a 3% to 10% increase in deproteinization rates. Alkaline ChCl (with a pKa value of 13.97) was used as the HBA, and the rate of deproteinization exhibited a positive correlation with the pKa value of the HBD at both 50 °C and 80 °C, which became more pronounced at 50 °C. However, when acidic Bet (pKa = 3.26) was employed as the HBA, the deproteinization rate positively correlated with the pKa of the HBD at 80 °C but negatively correlated at 50 °C. This suggests that deproteinization is more sensitive than demineralization to the pKa value of the HBD. The chemical method yielded α-chitin with a CrI of 38.82%, while black soldier fly prepupae had a CrI of 62.96%. All varieties of DES produced α-chitin with a lower CrI (31.34% to 50.76%) compared to chemical chitin or materials. This was found to have a positive correlation with the pH value of DES at 50 °C. The CrI was maximum at a pH of about 4.0 and declined for pHs greater or less than 4.0. Thus, the CrI of chitin produced by ChCl–LA DES (pH 0.16) and Bet–UR DES (pH 9.58) is the lowest. This could be because the presence of acidic HBAs or HBDs enhance the elimination of CaCO_3_, resulting in the cleavage of intra- and intermolecular hydrogen bonds and the formation of amorphous chitin.

Huet et al. [99] utilized ChCl–LA DES with a molar ratio of 1:2 to isolate chitin from larvae of domestic silk moth (*Bombyx eri*) and *Hermetia illucens*. The process involved a 1:50 S/L ratio, 110 °C for 2 h. The resulting chitin samples exhibited purity of 60.2% and 42.8% and recovery yields of 45% and 55% for *Bombyx eri* and *Hermetia illucens* larvae, respectively. The results were not optimal. Consequently, applying a low-concentration acid and alkali solution as a preliminary treatment, followed by DES treatment, increased the chitin purity for *Bombyx eri* and *Hermetia illucens* larvae by 3.5% and 5%, respectively. The ability of DES to dissolve amorphous chitin caused a 14% and 6% rise in CrI, respectively.

To summarize, Zhou et al. [115] obtained chitin with a purity of 91.34% from the skimmed powder of *Hermetia illucens* prepupae, whereas Huet et al. [99] produced chitin from the skimmed larvae of *Hermetia illucens* with a purity of just 42.8%. Despite variations in the S/L ratio and temperature employed during the isolation process, Huet et al. [99] hypothesized that employing a higher reaction temperature (110 °C versus 80 °C) and a higher S/L ratio (1:50 versus 1:10) would result in improved isolation efficiency based on previous research on other biomass. However, the actual outcomes diverged significantly from the anticipated results. The potential explanation is that the two studies employed distinct techniques for preparing the samples or utilizing different stages of the sample (larvae versus prepupae), which contain varying proportions of natural constituents [116]. In their study, Zhou et al. [115] highlighted that the pH value of DES has an inverse relationship with its demineralization capacity. However, it does not have a direct impact on product purity. In general, minerals and proteins are the two main contaminants that compromise the purity of chitin. Protein residues, in particular, are influenced by HBA acidity and HBD pKa value. Hence, the elimination of proteins is influenced by the pH of DES and regulated by the interaction between DES molecules or DES and proteins.

### 3.6. Mushrooms

Kim et al. [117] utilized five different forms of DES to isolate chitin–glucan complexes (CGCs) from Portobello mushroom (*Agaricus bisporus*). The isolation process was carried out in an S/L ratio of 1:20 by ultrasonic water bath for 1 h. The five types of DES were acidic ChCl–LA, Bet–LA, alkaline ChCl–UR, Bet–UR, and neutral ChCl–thiourea DES with a molar ratio of 1:2. The results showed that utilizing alkaline Bet–UR DES made it simpler to create CGCs with a lower yield (23.8%) and higher purity (20.5%) than acidic DES (ChCl–LA or Bet–LA DES). The capacity to remove proteins will vary depending on the specific DES utilized. Despite the chemical method having lower quantities of protein residue, alkaline Bet–UR DES exhibited the highest deproteinizing capability among the five types of DES. On the other hand, acidic DES exhibited the least ability to remove proteins. The use of alkali treatment is a well-established method for effectively eliminating proteins in the chitin production process. Strong alkalis can dissolve and degrade proteins, as well as weaken the covalent bonds between proteins and CGCs. Although ChCl–UR, ChCl–LA, and Bet–thiourea DES demonstrate greater efficacy in removing minerals than NaOH, deproteinization efficiency is the primary objective of isolating chitin from mushrooms. The Bet–UR DES CGCs with higher purity exhibited excellent deproteinization capability and were selected to investigate their properties further. The results were divided into supernatant and precipitate CGCs. The supernatant created by each form of DES had low yields, ranging from 2.2% to 4.5%. The precipitated CGCs exhibited a DA of 57.3% and a CrI of 37.0%, which are comparable to the values of 50.1% and 45.5% obtained through the chemical method. The supernatant of DA and CrI was 77.3% and 32.0%, respectively. These values suggest that chitin was the main constituent in the supernatant rather than chitosan. The low crystallinity of DES is due to its capacity for chitin dissolution.

Ozel and Elibol [118] examined four distinct techniques for isolating chitin and chitosan from *Agaricus bisporus*, using three different types of DES as solvents. ChCl–AA DES in a molar ratio of 1:2 was used in four different procedures: microwave-assisted for 3 min, ultrasonic-assisted at 55 °C for 2 h, and shaking water baths at 75 °C or 95 °C for 2 h. According to the results, microwave-assisted isolation produced the highest deproteinization rate of 38.7%, followed by 17.2% with shaking water bath at 95 °C. Consequently, the researchers opted to continue examining microwave-assisted isolation. The isolation procedure utilized ChCl–AA in a molar ratio of 1:2, ChCl–LA in a molar ratio of 1:1, and ChCl–GY DES in a molar ratio of 1:2. The deproteinization rate increased across all groups as the microwave time extended from 5 to 9 min, with ChCl–AA DES showing no significant change. Nonetheless, the rate of deproteinization increased since the S/L ratio increased from 1:5 to 1:20. Finally, microwaving for 9 min at an S/L ratio of 1:20, ChCl–AA DES showed the highest deproteinization rate of 84.25% and DA of 69%. Compared to commercial chitin (MW 250 kDa, CrI 80%), α-chitin isolated with ChCl–AA DES exhibited a lower MW of 120 kDa and CrI of 70%. In addition, ChCl–LA DES efficiently isolated α-chitin with a DA of 46%, indicating the formation of chitosan.

When employing DES to isolate CGCs or chitin from *Agaricus bisporus*, the particular DA or CrI chitin could be chosen by utilizing various DES compositions and distinct separation techniques. Kim et al. [117] discovered that the most effective protein removal occurred when alkaline Bet–UR DES were combined with an ultrasonic water bath for 1 h. Nonetheless, the results were less efficient than typical alkali chemistry procedures. Ozel and Elibol [118] found that microwave support for 3 min was more effective than ultrasonic aid for 2 h. When using acidic ChCl–AA DES, the protein removal rate could be as high as 84.25%. In the future, alkaline DES may be used in concert with microwave irradiation of mushroom-derived CGCs to produce physicochemical properties comparable to those obtained using standard chemical methods. However, further research is needed to evaluate this option entirely.

### 3.7. Squid Pens

McReynolds et al. [119] employed six distinct DES with varying pH levels, including acidic ChCl–MO and ChCl–LA, neutral Bet–GY and ChCl–UR, and alkaline Bet–UR and K_2_CO_3_-GY DES, to separate chitin from European squid (*Loligo vulgaris*) pens. The process was carried out at an S/L ratio of 1:25, with temperatures ranging from 50 °C to 120 °C for 2 or 3 h. The control was a traditional chemical process for chitin isolation, which yielded 32.3% ± 1.2% of chitin. To produce β-chitin with characteristics similar to those from chemical process, acidic ChCl–LA and neutral ChCl–UR DES were employed at 100 °C or 120 °C for 3 h. On the other hand, alkaline DES, such as Bet–UR and K_2_CO_3_–GY, can produce chitin similar to chemical β-chitin under various temperature and time conditions. The yield ranged from 31.5% to 34.9%, with K_2_CO_3_–GY DES being especially comparable. Furthermore, unlike the neutral ChCl–UR DES, which generated acetylated chitin at 120 °C for 3 h, alkaline DES did not acetylate chitin at high temperatures for an extended period. However, another study found that alkaline DES may deacetylate chitin [76]. Alkaline DES chitin had a lower DA than chemical chitin, where the DA of chitin obtained via chemical method was 97.5%. The DA of chitin generated by the K_2_CO_3_–GY DES in a molar ratio of 1:5 ranged between 77.6% and 88.6% at different temperatures and durations. In addition, the CrI of chitin derived from K_2_CO_3_–GY DES chitin varied between 88.3% and 91.2%, surpassing the CrI achieved using the chemical method (84.3%). This may be due to the ability of DES to eliminate amorphous chitin from the system at higher temperatures.

Sulthan et al. [120] combined Indian Ocean squid (*Uroteuthis duvaucelii*) pen powder with ChCl–MO DES in an S/L ratio of 1:25. The study found that using a 1:2 molar ratio at 80 °C for 2 h resulted in the highest β-chitin yield (42.76%).

Lv et al. [121] examined the performance of K_2_CO_3_–GY DES in varied molar ratios (ranging from 1:4 to 1:20) on separating β-chitin from squid pens at a 1:20 S/L ratio and 80 °C for varying durations (2 to 6 h). Research had verified that K_2_CO_3_–GY DES may establish hydrogen bonds through the oxygen atoms in K_2_CO_3_ and the hydroxyl groups in GY. As the quantity of K_2_CO_3_ increased, the intensity of the hydrogen bond contact force also increased. Augmenting the ratio of GY will bolster DES’s thermal stability and fluidity while diminishing DES’s viscosity. The findings indicated that the yields, demineralization, and deproteinization rate of β-chitin were comparable to those achieved using chemical methods, provided that the molar ratio of DES exceeds 1:7. Prolonging the duration of treatment will impact the pace of demineralization and deproteinization improvement. Nevertheless, there was no discernible disparity between 4 h and 6 h. A K_2_CO_3_–GY DES with a molar ratio of 1:10 was chosen for isolation at 80 °C for 4 h. The mineral and protein removal amounts were 1.10% and 67.30%, respectively, comparable to the rates achieved with a 4 h treatment with NaOH. Furthermore, the product yield reached 31.6%, comparable to the 28.2% achieved by chemical alkali treatment. The DA ranged from 64.2% to 69.7% in K_2_CO_3_:GY molar ratios ranging from 1:4 to 1:20, all less than the 78.6% observed with NaOH treatment. This indicated that the portion of K_2_CO_3_ did not influence the DA value. The CrI reached a maximum value of 81.2% when employing K_2_CO_3_–GY DES with a molar ratio of 1:10. This suggested that using the optimum K_2_CO_3_:GY molar ratio can stabilize the hydrogen bonding network and accelerate dissolution, resulting in the efficient removal of proteins and amorphous chitins. When the molar ratio was adjusted to 1:20, the CrI was 69.7%, closely matching the 65.2% obtained by the chemical method.

Alkaline DES effectively isolated β-chitin from squid pens. While acidic ChCl–MO DES exhibited some preparation efficiency [120], alkaline DES had a higher purity than chemical methods. It does not undergo the typical acylation reaction following treatment with acidic or neutral DES [119]. A frequent reaction in an alkaline environment is the deacetylation of chitin [76], which explains why the DA of chitin isolated by K_2_CO_3_–GY DES was lower compared to chemical methods [121]. Typically, an increase in alkaline content leads to more intense deacetylation. Nevertheless, the DA of chitin, produced via K_2_CO_3_–GY DES, was unaffected by the concentration of K_2_CO_3_. The contact force of the DES hydrogen bond network may be influenced by the ratio of K_2_CO_3_ to GY. According to Lv et al. [121], the hydrogen bonding interactions could be enhanced when applying a more significant amount of K_2_CO_3._ When the molar ratio of K_2_CO_3_ to GY was 1:10, the proton electron density in the GY hydroxyl group increased. This rise theoretically weakens the hydrogen bond interaction force of the DES system. Chitin with a high CrI (81.2%) could only be made under these specific conditions, surpassing the CrI achieved by traditional chemical methods (65.2%). Furthermore, an elevated temperature readily disrupts the hydrogen bonds between molecules [122]. Thus, when K_2_CO_3_–GY DES were subjected to a reaction at a molar ratio of 1:5 at 120 °C, its CrI (91.2%) was found to be higher compared to the chemical method (84.3%) [119]. This phenomenon occurs due to the weakening of DES’s hydrogen bond interaction force. As a result, the amorphous region of chitin was dissolved by DES, leading to an observed increase in crystallinity [115]. If the hydrogen bond interaction of DES becomes excessively strong, DES will destroy the crystalline region of chitin, causing a reduction in crystallinity. Nevertheless, additional study is required to provide further support.

### 3.8. Preparation Efficiency Elucidation

To better understand the efficiency of chitin preparation using DES, indicators such as yield, purity, demineralization and deproteinization efficiency were selected for comparison, with chemical methods serving as the standard. As shown in Table 1, DES compositions typically use ChCl as the HBA and organic acids as the HBD, with LA being the most common HBD. Chitin can be isolated from various crustacean exoskeletons (lobster, crab, shrimp, and crayfish shells) and even insects by varying the molar ratio. The purity of chitin obtained by DES showed only a slight difference (0.1% to 3%) compared to the chemical method, while the yield could increase by up to 13%. This improvement may be attributed to the effectiveness of LA as the HBD in purifying chitin and the lower viscosity of LA-based DES, which plays a crucial role in scalability and process efficiency. Low viscosity enhances reactivity and operability. Moreover, the recyclability of ChCl–LA DES has been confirmed in multiple studies. For example, Bradic et al. [97] observed a slight reduction in yield and purity after two recycling cycles, while Zhou et al. [115] found no significant difference in chitin purity after two cycles. Wang et al. [110] maintained high purity (92.71%) after three cycles of chitin preparation with ChCl–LA DES. McReynolds et al. [108] noted an increase in nitrogen content and a decrease in purity after three cycles of recycling, but Li et al. [113] showed that increasing the molar ratio of ChCl–LA DES to 1:10 and using a microwave-assisted process allowed up to four cycles, maintaining high purity (98.89% to 99.65%) and yields of 20.07% to 23.91%. Additionally, K_2_CO_3_–GY DES were most suitable for chitin preparation from squid pens, with yields similar to those obtained by the chemical method. The recyclability of K_2_CO_3_–GY DES has also been demonstrated; McReynolds et al. [119] showed that it could be recycled three times with no significant changes in yield or stability, while Lv et al. [121] observed consistent yields of around 31.5% per cycle after three cycles of recycling with a 1:10 molar ratio.

The recyclability of DES enhances the sustainability and economic value of this method. A preliminary comparison suggests that ChCl–LA DES are preferred for chitin resources with higher mineral content, such as crustaceans and black soldier flies. The molar ratio can range from 1:1 to 1:10, with increased LA proportion slightly improving purity. At a molar ratio of 1:2, the purity was comparable to that of chemical methods (less than 1% difference), and yields increased significantly (10% to 16%). For chitin resources with higher protein content, like squid pens, K_2_CO_3_–GY DES are recommended. The molar ratio can range from 1:5 to 1:10, yielding results similar to those of the chemical method. However, increasing GY content reduces viscosity [121] and improves operability, with a 1:10 molar ratio being the preferred option.

## 4. Mechanism of Chitin Preparation from Different Resources Using DES

**Figure 2:**
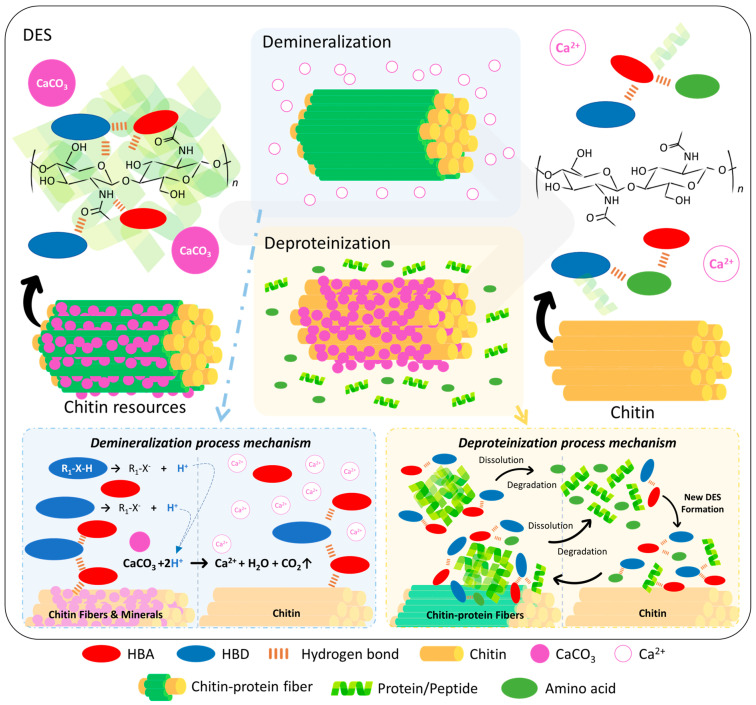
Schematic illustration of chitin separation by using deep eutectic solvents.

Numerous studies delineate the optimal conditions for DES chitin separation. Table 2 reviews the optimal DES ratios and preparation conditions employed in various investigations, enhancing comprehension of the process and facilitating further investigation into DES chitin separation methods. In addition, the composition of the raw materials significantly impacts chitin production. Although chitin’s properties and content vary with the season and growth stage [23], it remains a critical foundation. Table 3 depicts the basic composition of each raw material in this review. Crustaceans, including lobsters, shrimps, crabs, and crayfish, possess a significant mineral content, with an ash content ranging from 28.5% to 64.8% and a protein content between 8.1% and 36.47%. Nonetheless, non-crustaceans, including insects and mushrooms, possess a limited quantity of minerals and proteins, ranging from 0.88% to 5.54% and 2.8% to 32.59%, respectively. Squid pens comprise a substantial protein content of 50% to 70% and a minor mineral content of 1% to 5%. The choice of DES for the efficient separation of chitin was evident in the nature of the raw materials. Table 4 and Table 5 delineate the chitin properties isolated via DES from crustacean biomass, characterized by high mineral content, and non-crustacean biomass, characterized by low mineral content. Crustaceans predominantly utilize acidic HBDs or acidic DES, whereas non-crustaceans are not restricted to acidic DES alone. The elimination of minerals mostly depends on the release of H^+^ from organic acids to interact with CaCO_3_, whereas the removal of proteins is contingent upon the hydrogen bond network of DES, as illustrated in Figure 2. Moreover, many studies have been undertaken to elucidate the mechanisms by which DES facilitate the demineralization and deproteinization of chitin production, as illustrated in Table 6. The separation procedures are presented in succession according to different biomass resources.

### 4.1. α-Chitin from the Lobster Shells

Lobster shells comprise roughly 40.64% ash (CaCO_3_) and 25.83% protein [92]. Protein and chitin form protein–chitin fibers in which CaCO_3_ is embedded. Consequently, the elimination of minerals and proteins is crucial in the separation of chitin. Zhu et al. [94] assert that LA is crucial to demineralization. The ChCl–LA-based DES facilitated demineralization by the release of H^+^. Viscosity is essential for deproteinization. As the number of hydrogen bonds in the DES system escalated, the viscosity of DES rose, preventing its penetration into the lobster shells and resulting in partial demineralization. Conversely, removing a portion of CaCO_3_ increased the space among chitin–protein fibers, facilitating protein extraction from the chitin–protein fibers via the hydrogen bond network of DES. Furthermore, due to the abundance of carboxyl and hydroxyl groups, the protein can function as a new HBD, competing with Cl^−^ through electrostatic interactions to release H^+^. Ultimately, the protein will establish a new hydrogen bond with ChCl, disrupting the hydrogen bond between chitin–protein fibers to isolate the chitin.

### 4.2. α-Chitin from the Shrimp Shells

Shrimp shells comprise approximately 20% to 50% CaCO_3_, 20% to 40% protein, 15% to 40% chitin, and 0% to 14% lipids and pigments. The content of the substance can differ according to the species, season, and habitat in which it is found [126]. The composition variations of the shrimp shell reported in this review was comparable to that shown in Table 3. Chitin and protein combine to form chitin–protein fibers through inter- or intramolecular hydrogen bonding [127]. The rigid structure of shrimp shells is formed by the deposition of CaCO_3_ around the fibers. Huang et al. [103] first suggested that the crucial factor for separating chitin using ChCl–MA DES is the presence of acid. MA will initially attack the CaCO_3_ layer that covers the chitin–protein fibers, thereby exposing them. When DES come into contact with chitin–protein fiber, it will compete with and disturb the existing hydrogen bonds, creating new bonds between DES and chitin. Inter- or intramolecular hydrogen bonding can cause chitin to dissolve and separate it from proteins.

According to Feng et al. [96], the demineralization capacity of the organic acid employed in DES is determined by its pH value. The acid releases H^+^, which is then combined with CaCO_3_ to produce CO_2_ and calcium salts that dissolve in water. The content and molar ratio of organic acids determine the ability for removing proteins. Using ChCl as an example, when forming DES with MA, CA, or LA, the -COOH group of ChCl–MA DES will have a higher propensity to form hydrogen bonds with proteins. The proteins present in shrimp shells undergo a process of degradation into amino acids or water-soluble proteins. These converted proteins are then dissolved in solvents and eliminated. Thus, DES with a lower pH value has a more effective protein removal effect. Zhao et al. [104] employed a two-step approach to separate chitin from shrimp shells. Prior to processing, CA was employed to remove minerals from shrimp shells. They hypothesized that DES may eliminate proteins by establishing hydrogen interactions with chitin–protein fiber in shrimp shells. The network connecting chitin and protein in the shrimp shells is weakened, destroying the original hydrogen bonding network and the internal structure of the shrimp shells. The chitin is evenly distributed in DES and then isolated from the protein. Bradic et al. [97] stated that DES can dissolve chitin and provide a separation effect. The initial stage involves removing the protein tightly bound to the chitin fiber. Thus, subjecting the protein to high temperatures to denature it while simultaneously employing acidic or alkaline conditions to facilitate its hydrolysis into amino acids is imperative. The H^+^ of DES chemically reacts with the amine group of chitin, forcing the strong hydrogen bonds between and within chitin molecules to be disrupted. The next step is to remove minerals. The reaction between CaCO_3_ and the acidic component of DES results in the formation of calcium salts, water, and CO_2_. Ultimately, the chitin is dissolved in DES and separated from the shrimp shells.

Sun et al. [100], by real-time monitoring of variations in Ca^2+^ concentration, reported that shrimp shells treated with ChCl–LA DES and pure LA displayed comparable patterns and reached equilibrium after half an hour. Furthermore, the ChCl–LA DES have a pH value of 0.88, indicating the H^+^ concentration of 0.13 M. This allows the ChCl–LA DES to manage a reaction with CaCO_3_, resulting in the production of CO_2_ and water-soluble calcium salts. When utilizing the VMD 1.9.3 software to depict the distribution of electrostatic potential, it was seen that the electrostatic potential at the chlorine site in ChCl was at its lowest, thus validating its role as an HBA. Conversely, the hydrogen atoms of the carboxyl and hydroxyl groups in LA were highly noticeable. The interaction between ChCl and LA promotes the creation of many hydrogen bonds. The presence of a significant quantity of hydrogen bonds is a crucial factor in the capability of DES to dissolve proteins in shrimp shells. This is due to the formation of ammonium salts through the H^+^ from ChCl–LA DES and the proteins facilitated by electrostatic forces. As a result, a substantial amount of chlorine and lactic acid is attracted, forming new hydrogen bonds. A novel DES system was developed to disrupt the covalent bond between the protein and chitin in the shrimp shells by promoting the formation of competing hydrogen bonds. Ultimately, the protein was solubilized in ChCl–LA DES and separated from the chitin. As the reaction duration rose, there was no notable disparity in the residual mineral content, whereas the residual protein content diminished with increasing time. This further demonstrated that in the ChCl–LA DES, the LA component will initially bond with CaCO_3_, while the remaining LA will react with ChCl to produce DES. DES will then combine with protein to aid in the chitin purification process effectively. Following 3 h of processing, the protein residue remains consistently below 1%. The presence of sugar residues from chitin in shrimp shells and the presence of tyrosine or other α-amino acids in proteoglycans may contribute to the challenges encountered in isolating these substances.

Zhang et al. [101] investigated the separation of chitin from shrimp shells using ChCl–GY DES (with a molar ratio of 1:2) and a 7.5% concentration of AA. It has been verified that acid is not only crucial for identifying demineralization, but it also impacts the ability to remove proteins. In theory, 1 g of shrimp shell powder contains 0.4 g of CaCO_3_ and needs 0.6 g of AA to react with it. On the other hand, only 2.5% AA, represents 0.8 g of AA, can be sufficient to react with the minerals present in the shrimp shell powder. Nevertheless, a more effective demineralization effect was achieved when utilizing a 7.5% concentration of AA, suggesting that the presence of acid is a significant component influencing DES deproteinization. Feng et al. [98] employed acidic ChCl–TsOH DES with various molar ratios (3:1, 2:1, 1:1, 1:2, and 1:3) to separate chitin from shrimp shells. They also determined the pH values for each of these experimental groups. The readings were all approximately 1, suggesting that the released H^+^ concentrations are similar to each other, and both demonstrate strong demineralization capacity. Nevertheless, with an increasing quantity of TsOH, the residual protein content of precipitated chitin steadily diminished. The ChCl–TsOH DES exhibited the lowest protein content when the molar ratio was 1:1 since the deproteinization capacity is influenced by the molecule size and the position of hydrogen bonding after the composition of DES. He et al. [105] chose a novel MLA–MU–AA DES for isolating chitin from shrimp shells. They hypothesized that AA, acting as the hydrochloric acid in a chemical method, would undergo a reaction with CaCO_3_ and efficiently eliminate minerals present in shrimp shells. During the process of deproteinization, DES disrupt the bond between chitin and protein, facilitating the separation of protein from the chitin–protein fiber. AA further hydrolyzes the protein, allowing it and derived amino acids to dissolve into the DES system through hydrogen bonds, resulting in protein removal.

**Table 4:** The indices of chitin isolating process by deep eutectic solvents from crustaceans and the characteristics of obtained chitin.

Resources	HBA	HBD	Molar Ratio (HBA:HBD)	Yields (%)	Purity (%)	DM (%)	DP (%)	Mw (kDa)	DA (%)	CrI (%)	Ref.
Lobster shells	Choline chloride	Malonic acid	1:2	22.21	93	-	-	312	94.33	79.82	[93]
				16.19	-	-	-	-	-	80.6	[92]
		Lactic acid + Glycerol	1:1:1	26.22	94.76	98.77	95.99	655	-	77.73	[94]
Shrimp shells	Betaine HCl	Urea	1:2	23.6	-	98.15	93	330	92.2	70.8	[104]
	Choline chloride	Citric acid	1:3	45.85	86.44	-	-	-	-	99.01	[102]
		DL–Malic acid	1:2	27.2	92.6	-	-	-	-	94.1	[96]
			1:3	49.35	83.08	-	-	-	-	95.13	[102]
		Ethylene Glycol	1:2	24.8	-	98.15	90.6	340	93.4	80.8	[104]
		Glycerol	1:2	22.5	-	98.15	88.6	290	91.3	69.5	
				27.4	96.1	-	-	228	≈85	84.3	[101]
		Tartaric acid	1:3	48.85	87.73	-	-	-	-	96.31	[102]
		Lactic acid	1:1	20	98	-	-	125	89	91	[97]
			1:1	52.05	82.76	-	-	-	-	97.33	[102]
			1:2.5	-	99.33	-	-	-	-	80.32	[100]
		Malonic acid	1:2	23.86	-	-	-	79	107.78	87.59	[95]
		TsOH	1:2	59.4 ^R^	97.9	-	-	-	-	90.6	[98]
		Urea	1:2	25.1	-	98.15	92.0	370	95.1	81.0	[104]
	*N*-Methylacetamide	*N*-Methylurea, Acetic acid	1:1:3	-	-	96.74	94.29	837	93.23	82.83	[105]
Crab Shells	Choline chloride	Lactic acid	1:1	-	98.2	99.7	100.0	-	98.5	82.9	[79]
		Lactic acid + Glycerol	1:1:1	34.27	96.55	-	-	541	-	82.51	[110]
			1:1.5:0.5	36.75	96.53	-	-	382	-	83.62	
		Lactic acid	1:2	33.97	96.63	-	-	264	-	82.46	
				12.8	-	-	-	-	94.1	81.9	[108]
		Malic acid + Glycerol	2:1:1	34.38	95.69	-	-	239	-	84.79	[110]
			2:1.5:0.5	32.15	96.52	-	-	278	-	87.27	
		Malic acid	1:1	33.95	96.48	-	-	204	-	85.14	
				-	-	99.8	92.3	-	-	75.55	[106]
		Malonic acid	1:2	12.0	-	-	-	-	73.1	71.6	[108]
		*N*-acetyl-D-Glucosamine + Formic acid	1:0.6:1.4	85.6 ^R^	90.2	-	-	392	-	52.6	[82]
	Cystine	Gluconic acid	5:1	71.3 ^R^	94.1	-	-	375	-	74.9	[107]
	TEBAC	Lactic acid	1:27	21.31	91.15	97.36	88.94	-	86.20	78.85	[111]
Crayfish shells	Betaine	Lactic acid	1:2	18	85	-	-	224	96	85	[112]
	Choline chloride	Lactic acid	1:2	18	85	-	-	194	93	83	
			1:10	19.11	97.44	-	-	-	-	86.16	[113]
	Proline, Urea/75% DBU		1:2	89.23 ^R^	91.39	3.98	97.31	236	65	75.85	[114]

DBU: 1,8-Diazabicyclo [5.4.0] undec-7-ene; TEBAC: Triethylbenzylammonium chloride; TsOH: p-Toluenesulfonic acid monohydrate. ^R^: Recover yield%, Yield% = (mass of obtained precipitate (g) × purity%)/(mass of initial shrimp shells (g) × chitin%) × 100%.

### 4.3. α-Chitin from the Crab Shells

Crab shells and shrimp shells belonging to crustaceans primarily contain crystalline CaCO_3_ minerals. Table 3 displays the fundamental components of the three crab shell species addressed in this review. The crab shells had a significant proportion of acid-soluble chemicals, specifically 83.4% ± 1.1%, mostly minerals. By contrast, the remaining fraction of alkali-soluble substances was rather low, accounting for only 4.7% ± 0.6% of the exoskeleton [108]. Huang et al. [106] asserted that the primary obstacle was the demineralization in separating chitin from crab shells using eco-friendly DES. HBA–MA DES could eliminate minerals and expose the fragile chitin–protein fiber structure. DES possess the ability to disrupt hydrogen bonds and strong ionic strength interactions. Cl^−^ and -OH engage in competition to establish hydrogen bonds, resulting in the breakdown of the robust hydrogen bond network structure found in chitin–protein fibers. As a result, the protein became soluble in DES, facilitating the efficient separation of chitin. According to Wang et al. [107], the low acidity of DES contributes to demineralization. A substantial quantity of CO_2_ bubbles was promptly produced upon adding GA–Cys DES to crab shells, and the presence of free Ca^2+^ was observed in the solution. Concerning the deproteinization capacity, the identification of 16 soluble amino acids in the solvent verified that the proteins in crab shells were transformed into soluble proteins via weak acid treatment and degraded into soluble amino acids.

### 4.4. α-Chitin from the Crayfish Shells

Crayfish shells comprise 38.48% CaCO_3_, 21.84% protein, and 16.55% chitin, as seen in Table 3. Li et al. [113] used ChCl–LA DES to isolate chitin from crayfish shells. The demineralization reaction was attributed to acidic DES releasing H^+^ and reacting with CaCO_3_ to produce calcium salts, water, and CO_2_. The deproteinization reaction occurred through two interaction forces between protein and DES. These forces involved the formation of new hydrogen bonds between DES and protein, as well as the breaking of the amide band. Additionally, the amine salt of the protein and the Cl^−^ of DES easily formed electronic interactions. As a result, ChCl–LA DES not only weakened the inter- and intramolecular hydrogen bonds of chitin and proteins but dissolved proteins, effectively achieving chitin separation. Zhang et al. [114] developed a ternary Pro–UR/75% DBU DES demonstrating exceptional capability to remove proteins. DBU, a natural alkaloid, creates alkaline DES combined with proline and urea. This property facilitated the efficient dissolution of proteins. Furthermore, DBU has a high proton-grabbing capacity, allowing it to capture protons from the carboxyl group of proline. This leads to the accumulation of a negative charge, which facilitates penetration into chitin molecules at 110 °C. In addition, urea’s tiny molecular structure allows it to move readily under high temperatures. Consequently, intermolecular hydrogen connections are destroyed, allowing chitin to create new hydrogen bonds with other molecules. Pro–UR/75% DBU DES were utilized to dissolve chitin for protein removal, followed by applying 20% LA to eliminate minerals and separate the chitin.

**Table 5:** The indices of chitin isolating process by deep eutectic solvents from non-crustaceans and the characteristics of obtained chitin.

Resources	HBA	HBD	Molar Ratio (HBA:HBD)	Yields (%)	Purity (%)	DM (%)	DP (%)	Mw (kDa)	DA (%)	CrI (%)	Ref.
Prepupae	Betaine	Urea	1:1	12.01	90.52	-	-	-	95.59	≈44	[115]
	Choline chloride	Lactic acid	1:2	16.40	91.34	≈98	≈87	-	70.34	≈42.5	
Mushroom	Betaine	Urea	1:2	23.8	20.5	-	-	-	57.3	37.0	[117]
	Choline chloride	Acetic acid	1:2	-	-	-	84.25	120	69	70	[118]
Squid pens	Choline chloride	Malonic acid	1:2	42.76	-	-	-	-	-	-	[120]
	Potassium carbonate	Glycerol	1:5	31.5	-	-	-	31.5	77.6	91.2	[119]
			1:10	31.6	-	1.10 ^T^	67.30 ^T^	31.6	66.9	81.2	[121]

^T^: The minerals/proteins removal amounts of total squid pens weights.

### 4.5. α-Chitin from the Black Soldier Flies

The basic composition of the black soldier fly (*Hermetia illucens*) is influenced by various stages of growth, life cycles, and geographical regions [41,42]. Table 3 displays the basic composition of the two growth phases discussed in this review, including larvae and prepupae. The ash, protein, and chitin content ranges from 5.37% to 5.54%, 31.73% to 32.59%, and 8.40% to 9.74%, respectively. Other components, such as lipids and pigments, account for 21.62% to 26.63%. Zhou et al. [115] proposed that using ChCl–LA DES can effectively remove minerals (specifically CaCO_3_) from skimmed prepupae of black soldier flies. This process involved the release of H^+^ to eliminate the minerals, followed by the penetration of ChCl–LA DES into the protein–chitin fiber. DES then formed new hydrogen bonds with the protein, causing the existing hydrogen bonds of protein–chitin fiber to break. As a result, the chitin was separated from the protein. Although ChCl–UR DES had been found to dissolve 8% chitin [75], the protein exhibited more hydrophilic solubility than chitin. Proteins consist of numerous hydroxyl groups, carboxyl groups, and branched chains. These groups can function as an additional type of HBD, competing with Cl^−^ to create electrostatic interactions. This process induced the release of more H^+^, facilitating the hydrolysis of proteins into amino acids. Consequently, this leads to the effective removal of proteins.

### 4.6. α-Chitin from the Mushrooms

*Agaricus bisporus* mainly comprises 91% moisture, 0.88% ash, 2.8% protein, and 7.2% other components, as indicated in Table 3. The isolation of chitin from mushrooms often prioritizes the removal of proteins and often skips the demineralization step [128]. Ozel and Elibol [118] proved that DES could cause protein–chitin fibers to swell and separate proteins by disrupting the inter- and intramolecular hydrogen bonds. Because proteins contain more active functional groups, such as carboxyl, amine, and hydroxyl groups, which is more convenient for the HBA to establish new hydrogen bonds with proteins. Ultimately, hydrogen bonding existed within and between molecules of the chitin–protein fiber, which were destroyed, allowing the protein to be removed from the system.

### 4.7. β-Chitin from the Squid Pens

In general, dried squid pens from various species typically consist of approximately 25% to 40% chitin, 50% to 70% protein, and a minimal percentage (1% to 5%) of minerals. The minerals included in the sample consist primarily of CaCO_3_ with small quantities of sulfates [129]. Hence, the deproteinization process is important in preparing β-chitin from squid pens. Lv et al. [121] effectively employed an alkaline K_2_CO_3_–GY DES to isolate chitin from squid pens. A pure compound, K_2_CO_3_ or GY, was prepared with an equivalent concentration in K_2_CO_3_–GY DES to separate chitin under identical DES isolation conditions. However, many contaminants persisted in the resulting products, suggesting that chitin cannot be isolated using K_2_CO_3_ or GY. They suggested that alkaline DES should degrade and dissolve proteins by interacting with the two compounds to remove proteins. The DES compound forms hydrogen bonds with protein, destabilizing the link between β-chitin and proteins. As the GY percentage increased, the effectiveness of protein removal also increased, resulting in the release of more protein that can be dissolved into DES to generate new hydrogen bond interaction forces. Minerals in squid pens are dispersed throughout chitin–protein fibers and are eliminated as the protein degrades and dissolves. Thus, it also contributes to the elimination of minerals, particularly focusing on protein.

### 4.8. Mechanism Elucidation

When the raw material includes crustaceans (such as lobster, shrimp, crab, and crayfish), the typically utilized HBD is organic acid (Table 4). Research indicated that an increased number of carboxyl groups in the HBD structure correlates with a reduced pKa and lower pH value, facilitating greater H^+^ release and enhancing the separation effect [96,100,102,115]. Regardless of the selected HBA, the use of organic acids as HBDs could still lead to improved yields and purity [82,112]. Moreover, the application of alkaline or neutral DES, in conjunction with organic acids (including acetic, citric, and lactic acid), for a two-step chitin separation procedure demonstrated notable demineralization efficacy [101,104,114]. The demineralization mechanism was analogous to the traditional chemical method, wherein CaCO_3_ reacts with H^+^ to produce Ca^2+^ and CO_2_ in an acidic environment [82,103,106,113,130].

Regarding the deproteinization process, numerous studies have indicated that after demineralization, the protein–chitin fibers become exposed, enhancing the interaction between DES and the protein–chitin fibers. This interaction facilitates the further dissolution and degradation of the proteins, thereby either acidic or alkaline conditions hydrolyze them into amino acids [96,107]. Alternatively, a novel DES system could be developed to further enhance the solubilization of proteins within the chitin–protein fibers [94,98,113,114]. This effect may be attributed to the superior deproteinization capacity of alkaline DES, which, while effective for protein removal, shows limited demineralization efficiency. By contrast, acidic DES demonstrate both efficient deproteinization and demineralization [92,118,120]. The investigation on chitin separation using alkaline K_2_CO_3_–GY DES revealed that an increase in K_2_CO_3_ did not enhance the alkali treatment effect; rather, it was associated with an increasing proportion of glycerol. In addition, when the two compounds were used individually, they exhibited no impact [121]. The impact of deproteinization will be directly associated with the hydrogen bond interaction strength of DES [114,121]. Excessive hydrogen bonds in DES and elevated viscosity will hinder intermolecular mobility and diminish the separation efficacy [94]. Increased reaction temperature correlates with decreased viscosity and enhanced separation efficiency [79,93,107,108,111,113]. A prolonged reaction time facilitates the formation of additional hydrogen bonds between the proteins and DES, hence enhancing chitin separation efficiency [95,111,113,119,121]. Moreover, the efficiency of deproteinization may also be assessed based on the content of the raw materials. Mushrooms and squid pens, with a high protein content and a mineral content below 5%, were more effectively processed employing alkaline DES [117,119,120]. Prepupal shells possess a protein ratio comparable to other constituents; both alkaline and acidic DES had effective chitin separation features [115]. Nonetheless, for crustaceans with high mineral content (20% to 50%), the efficacy of alkaline DES are comparatively diminished, with acidic DES being the primary subject of investigation [92,93].

We assert that in the application of DES for the separation of chitin from raw materials, the steps of removing minerals and proteins are performed simultaneously and complement each other, as shown in Figure 2. Most chitin preparation methods involve demineralization followed by deproteinization, as the dissolution of CaCO_3_ increases surface area, hence facilitating subsequent protein degradation [12]. Nevertheless, certain research conducted deproteinization prior to demineralization and determined that altering the sequence of chemical processes did not substantially influence the quality and yield of chitin produced [131]. In those chitin raw materials, chitin–protein fibers embedded with CaCO_3_ were covered by DES and established partial hydrogen bonds with chitin. Following demineralization and deproteinization, the degraded proteins or amino acids will generate new forms of DES, disrupt the hydrogen bonds between DES–chitin and chitin–protein fibers, and isolate chitin from the raw materials.

In demineralization, the HBD in DES releases H^+^, which interacts with CaCO_3_, forming calcium salt, carbon dioxide, and water, thereby achieving the demineralization effect. Simultaneously, following the dissolution of proteins by DES, they will be degraded by alkali or acid into amino acids, which will then combine with the original DES to create a new DES–amino acids or DES–proteins system. The hydrogen bond network will be generated between the newly synthesized DES–amino acids/proteins, supplanting the initial hydrogen bond interactions between chitin–protein fibers and DES. This enables the initial DES to release more H^+^, which interacts with CaCO_3_ to remove minerals. Simultaneously, the freshly synthesized DES–proteins or DES–amino acids solubilize more proteins from chitin–protein fibers, facilitating their degradation into amino acids. This further stabilizes the hydrogen bond network of DES–proteins/amino acids, finally resulting in the hydrogen bonding force within the chitin molecule surpassing the contact force between chitin–proteins or chitin–DES, hence causing chitin to separate and precipitate out. This may also elucidate the presence of partially amorphous chitin. The hydrogen bond network in the amorphous chitin molecule is insufficiently strong, allowing it to establish hydrogen bonds with DES or DES–amino acids/proteins, making precipitation challenging; thus, it remains dissolved in DES. However, additional research is necessary. 

**Table 6:** Mechanism of chitin isolating with deep eutectic solvents.

Chitin Type	Resource	Proposed Mechanism		Ref.
		Demineralization	Deproteinization	
α-chitin	Lobster shells	Lactic acid was used to remove CaCO_3_. In addition, the elevated viscosity impedes the penetration of DESs into lobster shells, leading to incomplete removal of CaCO_3_ and consequently reducing the purity of the extracted chitin. Due to the partial removal of CaCO_3_ by DESs, the linkages in the inner structural organization of lobster shells were weakened.	Proteins are rich in carboxylic and hydroxyl groups, serving as HBD that compete for chloride anions through electrostatic interaction by inducing H^+^, resulting in most lactic acid being attracted. Hence, new hydrogen bonds were formed between choline chloride and protein, disrupting the hydrogen bonds within the protein–chitin fibers.	[94]
α-chitin	Shrimp shells	Malic acids carried out demineralization. The malic acid removed the shrimp shells and minerals, which are mostly in the form of crystalline CaCO_3_, leaving the proteins and chitin. The spacing between the chitin–protein fibers was filled with proteins and minerals; thus, removing minerals weakened the linkages within the inner structural organization of the shrimp shells.	Competing hydrogen bond formation between DES and carbohydrates breaks the intramolecular hydrogen bond network, weakening the shrimp shells’ hydrogen bond interactions. As a result, chitin is dissolved in DES and separated from the proteins.	[103]
α-chitin	Shrimp shells	The pH of DES was about 1.5, indicating that H^+^ can be released from DES. The released H^+^ reacted with calcium carbonate to produce CO_2_ and water-soluble calcium salts, removing CaCO_3_.	The protein in the shrimp shells in DES was degraded to amino acids or a water-soluble protein, which can be dissolved in DES to remove it.	[96]
α-chitin	Shrimp shells	The removal of minerals was mainly accomplished through the lactic acid of DES. The pH value of DES was about 0.88. The reaction of minerals in shrimp shells in the form of crystalline CaCO_3_ with H+ is released from DES to produce CO_2_ and water-soluble calcium salts.	The H^+^ present in DES forms ammonium salts with proteins by electrostatic forces that attract large amounts of chlorine and lactic acid, thus forming new hydrogen bonds. The appearance of competing hydrogen bonds weakened the covalent bond between the protein and chitin in the shrimp shell. The protein was dissolved into DES and separated from the chitin.	[100]
α-chitin	Crab shells	The weak acidity of reagents was the main driving force of demineralization. Many CO_2_ bubbles were rapidly generated when DES were added to the crab shells. More importantly, free Ca^2+^ was detected in the solution after DES treatment.	The protein in the crab shell was removed in two ways: one was converted into soluble protein, and the other was degraded into soluble amino acids. Sixteen common amino acids were present in the supernatant after DES treatment. Simultaneously, the content of nine amino acids increased after HCl treatment.	[107]
α-chitin	Crab shells	Because the minerals in crab shells are mostly in the form of crystalline CaCO_3_, when DES were applied to the crab shells, minerals were removed by malic acid, leaving chitin and protein. The strong internal structure of crab shells was weakened after removing minerals.	The strong hydrogen-bond network between chitin and proteins was weakened due to competing hydrogen bonds formed between the Cl^−^ of DES and the hydroxyl groups, and the proteins were removed by dissolution because of the hydrogen-bond interaction with DES.	[106]
α-chitin	Prepupae	The ability of the released H^+^ from the acidic solvents of DES was attributed to removing crystalline CaCO_3_. This decreased the linkages between protein and chitin, which facilitates the soaked behaviors between DES and protein–chitin fibers. The release of H^+^ was the key factor in the removal of minerals.	The new hydrogen bonds were generated between DES and the protein, which damaged the hydrogen bond formed in protein–chitin fibers. On the other hand, the amounts of peptide bonds hydrolyzed by the released H^+^ resulted in the formation of free amino acids during deproteinization.	[115]
β-chitin	Squid pens	The proteins and minerals were removed from squid pens through the synergistic action of alkaline DES formed by K_2_CO_3_ and glycerol. As a component of squid pens, minerals were distributed between chitin–protein fibers and removed with the degradation and dissolution of proteins. Hence, the alkaline DESs also played the role of demineralization.	Alkaline K_2_CO_3_ and glycerol played the role of protein degradation and dissolution, respectively, and the hydrogen bond interaction with DES further weakened the binding between ß-chitin and protein and increased protein dissolution. In addition, the efficient dissolution of proteins accelerated protein dissolution, when the protein removal efficiency was increased by increasing glycerol content.	[121]

It is important to reiterate that the demineralization and deproteinization of chitin, separated by DES, are complementary processes. That acid can efficiently extract minerals from raw materials, reveal a greater surface area for chitin–protein fibers, and facilitate partial acid hydrolysis of proteins. Neither acid nor alkali inhibits the dissolution of proteins by the hydrogen bond network of DES. Alkalines can more effectively solubilize proteins and degrade them into amino acids, enhancing the robust hydrogen bond network of DES and aiding purification by eliminating minerals associated with chitin–protein fibers. This suggests that the majority of crustaceans possessing elevated mineral content prefer DES characterized by both acidity and a robust hydrogen bonding network. Conversely, non-crustaceans with lower mineral content, including insects, mushrooms, and squid pens, are not restricted to acidic DES. Alkaline DES with a strong hydrogen bond network separated chitin from squid pens containing exceptionally high protein contents. However, an abundance of hydrogen bond networks will affect the viscosity of DES, reducing separation efficiency. Therefore, in conjunction with the hydrogen bond network of DES, we must also consider its viscosity.

## 5. Environmental Impact for DES Isolation Technology and Their Potential Practical Application

Although DES have been recognized as a promising green alternative to conventional preparation methods, their greenness and sustainability are not yet fully established and require rigorous quantitative evaluation. To date, no comparative or comprehensive studies have assessed the environmental impacts of DES relative to traditional chemical methods in chitin preparation from bio-waste.

Some individual studies suggest that the DES isolation process may have advantages in terms of water usage and wastewater output. For instance, Lopes et al. [132] reported that conventional chemical methods require water usage approximately 20 times the quantity of the raw materials (consuming 30,462 tons of water to process 1523.1 tons of crustacean biomass) and result in wastewater outputs 21.8 times that of the chitin produced (418.9 tons of chitin generating 9139 tons of wastewater). In comparison, the DES isolation process requires only about three times the amount of water relative to raw materials to wash residual DES from the chitin products, followed by a decolorization step using H_2_O_2_ [79], which results in significantly lower water deprivation potential (WDP). WDP is a key environmental impact indicator [133], used to inform life cycle assessment (LCA) methodologies and calculate Product Environmental Footprints (PEF). However, some laboratory-scale DES processes for chitin preparation, where decolorization was omitted, required much larger amounts of water (up to 90 times the raw material quantity) to remove residual DES and pigments, potentially causing a greater environmental impact than conventional chemical methods.

In conducting an LCA to assess the environmental impact of DES-based processes, it is essential to consider not only the washing steps but the environmental impacts of the DES chemicals used for preparation. A recent LCA study on ChCl–UR—a commonly used DES—revealed substantial environmental impacts, including a global warming potential (GWP) of 1.82 kg CO_2_-Eq/kg, freshwater eutrophication potential (FEP) of 0.000471 kg P-Eq/kg, terrestrial acidification potential (TAP) of 0.00688 kg SO_2_-Eq/kg, metal depletion potential (MDP) of 0.087 kg Fe-Eq/kg, water depletion potential (WDP) of 0.00478 m³/kg, freshwater ecotoxicity potential (FETP) of 0.0407 kg 1,4-DCB-Eq/kg, and human toxicity potential (HTP) of 0.517 kg 1,4-DCB-Eq/kg. These values surpass those for certain traditional solvents, such as methanol and ethanol. The study also compared environmental impacts across different DES, finding that ChCl–CA—a natural DES—had higher life cycle environmental impacts than other tested DES, such as ChCl–UR, ChCl–GY, and ChCl–ethylene glycol. This was partly attributed to high water and CO_2_ emissions during the citric acid fermentation process [134].

A separate study assessed DES for phenolic extraction from spent coffee grounds and reported that DES performed worse than ethanol 20% and water across all environmental impact categories. Here, the environmental impact of DES was mainly associated with the preparation of virgin raw materials and the adsorption step involving resins [135].

Therefore, it would be premature to conclude that DES preparation is inherently a green process. A comprehensive evaluation of DES types, preparation efficiency, and washing and decolorization methods is necessary to fully assess its greenness and sustainability. Additional research is also needed to confirm the environmental impacts of using DES for chitin isolation.

The potential for scaling up DES applications for chitin preparation and the feasibility of their industrial implementation remain underexplored, paralleling the gaps in understanding their environmental impact. We have identified five key limitations contributing to these challenges:

i. Lack of standardized processes:

DES are formulated from various combinations of HBAs and HBDs, each yielding different isolation efficiencies and product purities depending on the type of bio-waste used. Consequently, separation and purification procedures following extraction also vary. This review represents the first systematic summary of isolation performance across different DES types and raw materials, aiming to provide a robust foundation for future research in this area.

ii. Product purity constraints:

Chitin intended for high-value biomedical and other specialized applications generally requires a purity level above 99%. Among the studies reviewed, only one achieved a final product purity above 99% (99.33%) [100], while two others reached levels over 98% (98.2% and 98.0%) [79,97]. This indicates that most DES-derived products would still need further purification to meet market standards, underscoring that the current processes are not yet fully optimized and would benefit from further refinement in process parameters and unit operations.

iii. Residues in final products:

Limited studies on DES isolation have analyzed residues in the resulting chitin. DES components, along with residual metal ions and pigments, may persist in the chitin, potentially limiting its suitability for biomedical or food applications.

iv. High cost of chemicals and lack of cost-effectiveness evaluation:

Compared to traditional chemical preparation methods, DES rely on relatively costly chemicals. Although DES processes may offer cost advantages due to reduced water usage and wastewater treatment, there is a need for comprehensive cost-effectiveness analyses comparing DES with conventional methods. Such evaluation is essential before designing scalable processes.

v. Uncertain safety profile:

Typical DES formulations exhibit higher FETP and HTP than conventional solvents like methanol and ethanol (e.g., ChCl–UR: 0.04070 kg 1,4-DCB-Eq/kg vs. methanol: 0.01040 kg 1,4-DCB-Eq/kg and ethanol: 0.00237 kg 1,4-DCB-Eq/kg for FETP; ChCl–UR: 0.5170 kg 1,4-DCB-Eq/kg vs. methanol: 0.0858 kg 1,4-DCB-Eq/kg and ethanol: 0.1400 kg 1,4-DCB-Eq/kg for HTP) [134]. Some NADES exhibit even higher FETP and HTP values than ChCl–UR. Thorough safety assessments are essential to ascertain the feasibility of using DES on an industrial scale.

Therefore, the potential industrial application of DES-based chitin preparation will depend on further research into process optimization, environmental impact assessment, cost-effectiveness analysis, and comprehensive safety evaluation.

## 6. Conclusions

Deep eutectic solvents (DES) align with green chemistry principles, offering a more sustainable and forward-looking approach to chitin isolation compared to traditional chemical methods. Studies have consistently demonstrated the effectiveness of DES in isolating chitin from various sources, including lobster shells, shrimp shells, crab shells, crayfish shells, squid pens, insects, and mushrooms. The resulting chitin had comparable effectiveness and physicochemical properties to those obtained through conventional methods. When selecting the appropriate DES, the proximate composition of the raw materials is crucial. For materials with low mineral content and high protein levels, alkaline DES is recommended, whereas those with high mineral content should prioritize acidic DES. Organic acids in natural deep eutectic solvents (NADES) can facilitate the removal of calcium carbonate from biomass. The hydrogen bond network in NADES may help degrade proteins in the raw materials into amino acids and further dissolve them consequently. Furthermore, it has the ability to help dissolve amorphous chitin, hence improving the precipitated chitin’s crystalline structure. Moreover, NADES prevents excessive deacetylation and degradation of molecular weight caused by strong alkalis and can even promote the direct formation of acylated chitin during processing. By tailoring different types of NADES and preparation techniques, chitin with desired properties, such as specific molecular weights, acetyl degrees, or crystallinity, can be efficiently produced. The comparative environmental impact of the DES isolation process versus conventional chemical methods for chitin preparation remains insufficiently explored, leaving the sustainability and ‘greenness’ of the process unclear. A more comprehensive study is needed to establish foundational data that can inform future industrial scale-up applications.

## Figures and Tables

**Table 1:** Comparison of the efficiency between various DES and chemical methods for chitin preparation.

DES (HBA–HBD)	Molar Ratio (HBA:HBD)	Resources	DES Method				Chemical Method				Ref.
			Yields (%)	Purity (%)	DM (%)	DP (%)	Yields (%)	Purity (%)	DM (%)	DP (%)	
Bet–Acetic acid	1:2	Crab shells	71.2 ^R^	88.4	-	-	58.7 ^R^	95.6	-	-	[82]
Bet–Formic acid			70.1 ^R^	92.2	-	-	58.7 ^R^	95.6	-	-	
Bet–Levulinic acid			75.4 ^R^	88.7	-	-	58.7 ^R^	95.6	-	-	
Bet–Urea	1:1	Prepupae	12.01	90.52	-	-	6.5	91.63	-	-	[115]
	1:2	Mushrooms	23.8	20.5	-	-	17.0	26.5	-	-	[117]
ChCl–Levulinic acid		Lobster shells	20.23	91	-	-	17.21	93	-	-	[93]
ChCl–Acetic acid		Crab shells	71.5 ^R^	89.5	-	-	58.7 ^R^	95.6	-	-	[82]
ChCl–Acetyl-glucosamine + Formic acid	1:0.6:1.4		85.6 ^R^	90.2	-	-	58.7 ^R^	95.6	-	-	
ChCl–Formic acid	1:2		66.2 ^R^	93.4	-	-	58.7 ^R^	95.6	-	-	
ChCl–Lactic acid	1:1	Lobster shells	23.31	90	-	-	17.21	93	-	-	[93]
		Crab shells	-	98.2	99.7	100.0	-	-	100.0	100.0	[79]
	1:2	Shrimp shells	29.20	-	-	-	16.08	-	-	-	[95]
		Crab shells	12.8	-	-	-	12.9	-	-	-	[108]
		Prepupae	16.40	91.34	≈98	≈87	6.5	91.63	-	-	[115]
	1:2.5	Shrimp shells	-	99.33	-	-	-	99.55	-	-	[100]
	1:10	Crayfish shells	19.11	97.44	-	-	19.63	97.31	-	-	[113]
ChCl–Lactic acid + Glycerol	1:1:1	Lobster shells	26.22	94.76	98.77	95.99	16	98.23	99.06	99.17	[94]
ChCl–Levulinic acid	1:2	Crab shells	75.0 ^R^	90.3	-	-	58.7 ^R^	95.6	-	-	[82]
ChCl–Malic acid	1:1	Lobster shells	23.06	90	-	-	17.21	93	-	-	[93]
ChCl–Malonic acid	1:2		22.21	93	-	-	17.21	93	-	-	
ChCl–Malonic acid			16.19	-	-	-	16.53	-	-	-	[92]
		Shrimp shells	23.86	-	-	-	16.08	-	-	-	[95]
		Crab shells	12.0	-	-	-	12.9	-	-	-	[108]
Cystine–Gluconic acid	5:1		71.3 ^R^	94.1	-	-	58.7 ^R^	95.6	-	-	[107]
K_2_CO_3_–Glycerol	1:5	Squid pens	31.5	-	-	-	32.3	-	-	-	[119]
	1:10		31.6	-			28.2	-	-	-	[121]
Methylacetamide-Methylurea + Acetic acid	1:1:3	Shrimp shells	-	-	96.74	94.29	-	-	99.15	96.45	[105]

Bet: Betaine; ChCl: Choline chloride. ^R^: Recover yield%, Yield% = (mass of obtained precipitate (g) × purity%)/(mass of initial shrimp shells (g) × chitin%) × 100%.

**Table 2:** The optimal conditions for separating chitin from different sources using deep eutectic solvents.

Resources	HBA	HBD	Molar Ratio (HBA:HBD)	S/L	Temp. (°C)	Time	Ref.
Lobster shells	Choline chloride	Malonic acid	1:2	1:10	50	2 h	[93]
				1:14			[92]
		Lactic acid + Glycerol	1:1:1	1:20			[94]
Shrimp shells	Betaine HCl	Urea	1:2	1:20	After 10% CA & microwave	7 min	[104]
	Choline chloride	Citric acid	1:3	1:20	80	2 h	[102]
		DL–Malic acid	1:2	1:20	130	3 h	[96]
			1:3		80	2 h	[102]
		Ethylene Glycol	1:2	1:20	After 10% CA and microwave	7 min	[104]
		Glycerol	1:2	1:20	After 10% CA and microwave	7 min	
				1:29	100 (+7.5% AA at 120)	3 h (1 h)	[101]
		Tartaric acid	1:3	1:20	80	2 h	[102]
		Lactic acid	1:1	1:50	60	6 h	[97]
			1:1	1:20	80	2 h	[102]
			1:2.5		150	6 h	[100]
		Malonic acid	1:2	1:25	80	2 h	[95]
		TsOH	1:2	1:20	70	3 h	[98]
		Urea	1:2	1:20	After 10% CA and microwave	7 min	[104]
	*N*-Methylacetamide	*N*-Methylurea, Acetic acid	1:1:3	1:30	Room temp.	48 h	[105]
Crab Shells	Choline chloride	Lactic acid	1:1	1:25	130	2 ** h	[79]
		Lactic acid + Glycerol	1:1:1	1:20	80	2 h	[110]
			1:1.5:0.5				
		Lactic acid	1:2	1:20	80	2 h	
				1:25	120		[108]
		Malic acid + Glycerol	2:1:1	1:20	80	2 h	[110]
			2:1.5:0.5				
		Malic acid	1:1	1:20	80	2 h	
				1:30	Microwave	11 min	[106]
		Malonic acid	1:2	1:25	120	2 h	[108]
		*N*-acetyl-D-Glucosamine + Formic acid	1:0.6:1.4	1:20	130	3 h	[82]
	Cystine	Gluconic acid	5:1	1:20	100	6 h	[107]
	TEBAC	Lactic acid	1:27	1:20	120	6 h	[111]
Crayfish shells	Betaine	Lactic acid	1:2	1:20	115	20 h	[112]
	Choline chloride	Lactic acid	1:2	1:20	115	20 h	
			1:10	1:10	Microwave at 120	0.5 h	[113]
	Proline, Urea/75% DBU		1:2	1:20	110 (+20% LA at 50)	20 h (1 h)	[114]
Prepupae	Betaine	Urea	1:1	1:10	80	2 h	[115]
	Choline chloride	Lactic acid	1:2	1:10	80	2 h	
Mushrooms	Betaine	Urea	1:2	1:20	Ultrasonic	1 h	[117]
	Choline chloride	Acetic acid	1:2	1:20	Microwave	9 min	[118]
Squid pens	Choline chloride	Malonic acid	1:2	1:25	80	2 h	[120]
	Potassium carbonate	Glycerol	1:5	1:25	120	2 h	[119]
			1:10	1:20	80	4 h	[121]

AA: Acetic acid; CA: Citric acid; DBU: 1,8-Diazabicyclo [5.4.0] undec-7-ene; LA: Lactic acid; TEBAC: Triethylbenzylammonium chloride; TsOH: p-Toluenesulfonic acid monohydrate. **: After a certain period of DES isolation, H_2_O is added and mixed until reaching room temperature.

**Table 3:** Basic composition and specific species for chitin separation resources discussed in this review paper.

Resources	Moisture (%)	Ash (%)	Protein (%)	Chitin (%)	Others (%)	Ref.
Lobster shells	7.30 ± 0.05	40.64 ± 0.23	25.83 ± 0.85	-	-	[92]
Shrimp shells (*Marsupenaeus japonicus*)	12.56 ± 1.09	31.76 ± 8.17	36.47 ± 1.25	-	-	[95]
shrimp shells (*Solenocera crassicornis*)	-	56.1	8.1	35.8	-	[96]
Crab shells (*Cancer pagurus*)	-	64.8	-	11.4	23.8	[79]
Crab shells (*Polybius henslowii*)	-	44.5 ± 0.57	32.1 ± 6.68	9.7 ± 0.57	13.2 ±0.25	[123]
Crab shells (*Chionoecetes opilio*)	-	28.5	34.2	-	17.1	[124]
Crayfish shells	5.78	38.48	21.84	16.55	-	[114]
Larvae (*Hermetia illucens*)	6.30 ± 0.03	5.54 ± 0.01	31.73 ± 0.65	8.40 ± 0.20	21.62 ± 0.11	[41]
Prepupae (*Hermetia illucens*)	4.20 ± 0.16	5.37 ± 0.04	32.59 ± 0.11	9.74 ± 0.66	26.63 ± 0.37	
Mushrooms (*Agaricus bisporus*)	91	0.88	2.8	-	7.32	[125]

## Data Availability

The original contributions presented in the study are included in the article, further inquiries can be directed to the corresponding author.
